# Holospiniferoside: A New Antitumor Cerebroside from The Red Sea Cucumber *Holothuria spinifera:* In Vitro and In Silico Studies

**DOI:** 10.3390/molecules26061555

**Published:** 2021-03-12

**Authors:** Enas E. Eltamany, Usama Ramadan Abdelmohsen, Dina M. Hal, Amany K. Ibrahim, Hashim A. Hassanean, Reda F. A. Abdelhameed, Tarek A. Temraz, Dina Hajjar, Arwa A. Makki, Omnia Magdy Hendawy, Asmaa M. AboulMagd, Khayrya A. Youssif, Gerhard Bringmann, Safwat A. Ahmed

**Affiliations:** 1 Department of Pharmacognosy, Faculty of Pharmacy, Suez Canal University, Ismailia 41522, Egypt; enastamany@gmail.com (E.E.E.); dina_hal@pharm.suez.edu.eg (D.M.H.); am_kamal66@yahoo.com (A.K.I.); amahdali@gmail.com (H.A.H.); omarreda_70@yahoo.com (R.F.A.A.); 2 Department of Pharmacognosy, Faculty of Pharmacy, Deraya University, New Minia 61111, Egypt; usama.ramadan@mu.edu.eg; 3 Department of Pharmacognosy, Faculty of Pharmacy, Minia University, Minia 61519, Egypt; 4 Department of Marine Science, Faculty of Science, Suez Canal University, Ismailia 41522, Egypt; ttemraz@science.suez.edu.eg; 5 Department of Biochemistry, Collage of Science, University of Jeddah, Jeddah 80203, Saudi Arabia; dhajjar@uj.edu.sa (D.H.); amaki@uj.edu.sa (A.A.M.); 6 Department of Chemistry of Pharmacology, Faculty of Pharmacy, Jouf University, Skaka 2014, Saudi Arabia; omhendawy@ju.edu; 7 Department of Clinical Pharmacology, Faculty of Medicine, Beni Suef University, Beni-Suef 62513, Egypt; 8 Pharmaceutical Chemistry Department, Faculty of Pharmacy, Nahda University, Beni Suef 62513, Egypt; asmaa.aboulmaged@nub.edu.eg; 9 Department of Pharmacognosy, Faculty of Pharmacy, Modern University for Technology and Information, Cairo 12585, Egypt; khayrya.youssif@gmail.com; 10 Institute of Organic Chemistry, University of Würzburg, Am Hubland, 97074 Würzburg, Germany

**Keywords:** *Holothuria spinifera*, HRMS, cerebrosides, molecular docking, cytotoxicity

## Abstract

Chemical investigation of the methanolic extract of the Red Sea cucumber *Holothuria spinifera* led to the isolation of a new cerebroside, holospiniferoside (**1**), together with thymidine (**2**), methyl-*α*-d-glucopyranoside (**3**), a new triacylglycerol (**4**), and cholesterol (**5**). Their chemical structures were established by NMR and mass spectrometric analysis, including gas chromatography–mass spectrometry (GC–MS) and high-resolution mass spectrometry (HRMS). All the isolated compounds are reported in this species for the first time. Moreover, compound **1** exhibited promising in vitro antiproliferative effect on the human breast cancer cell line (MCF-7) with IC_50_ of 20.6 µM compared to the IC_50_ of 15.3 µM for the drug cisplatin. To predict the possible mechanism underlying the cytotoxicity of compound **1**, a docking study was performed to elucidate its binding interactions with the active site of the protein Mdm2–p53. Compound **1** displayed an apoptotic activity via strong interaction with the active site of the target protein. This study highlights the importance of marine natural products in the design of new anticancer agents.

## 1. Introduction 

Cancer is considered as the main cause of mortality and morbidity all over the world [1]. Therefore, there is an urgent need for the discovery of new anticancer agents with new mechanisms of action. Natural sources such as microbes, plants, and marine organisms are regarded as a huge reservoir of novel bioactive metabolites [2,3]. Numerous marine-derived compounds act as antineoplastic drugs via growth inhibition of human carcinoma in vitro and in vivo [4,5]. The immense studies on marine metabolites have resulted in the discovery of a novel generation of antineoplastic drugs that have entered the clinical trial phase [6]. Sea cucumbers (Holothuroidea class) are considered as one of the most abundant marine taxa. They include about 1400 species of six orders worldwide (Apodida, Aspidochirotida, Elasipodida, Molpadiida, Dendrochirotida, and Dactylochirotida) and 25 families [7]. Sea cucumbers are present in all oceans and also in shallow waters. They are consumed worldwide as food and in folk medicine, where their extracts have gained massive popularity among nutritionists and researchers for their high nutritive and therapeutic values. Sea cucumber extracts have suppressed inflammation and increased innate immune responses [8]. One of the most important diverse families of sea cucumbers is the Holothuriidae. This taxon comprises five genera [9]. Genus *Holothuria*, is the most predominant genus reported in the World Register of Marine Species database [10], is represented in Egypt by eight species [11]. There are several reports on the bioactivities of *Holothuria* species. The organic extracts of the sea cucumber species (*Holothuria leucospilota* and *Holothuria scabra*) exert antiproliferative action on human C33A cervical cancer cells and A549 nonsmall lung cancer cells [8]. In addition, *Holothuria spinifera* extract possess anticancer properties against the breast cancer cell line (MCF-7) [12]. Moreover, *Holothuria leucospilota* have antimicrobial and antileishmanial effects [13,14]. Furthermore, *Holothuria scabra* has been reported to have high antioxidant capacity [15]. Esmat et al. have reported the antioxidant and hepatoprotective activities of *Holothuria atra* against thioacetamide intoxication [16]. These unique biological activities are correlated to the diverse chemical entities in *Holothuria* species. Phytochemical investigation of *Holothuria* sea cucumbers has led to the isolation and identification of numerous bioactive constituents of diverse chemical classes [17,18]. Several glycosphingolipids, ceramides, triterpene glycosides, and esterified phospholipids have been reported in the genus *Holothuria*. Besides, this genus is rich in minerals, vitamins, and peptides and also contains unique molecules, such as 12-methyltetradecanoic acid (12-MTA), philinopside E, glycosaminoglycan, and chondroitin sulfate [19]. These compounds have been reported to possess anticancer, antiangiogenic, antimicrobial, anti-inflammatory, and immunomodulatory properties [7]. It is worth noting that we have previously reported the isolation of new cytotoxic cerebrosides [12]. Hence, the present study aims to pursue our phytochemical investigation of *Holothuria spinifera*, assess the antitumor effect of the newly isolated compounds against the breast cancer (MCF-7) cell line, and predict the possible mechanism of action by the aid of molecular-docking approaches.

## 2. Results and Discussion

### 2.1. Structure Elucidation of the Isolated Compounds

**Figure 1:**
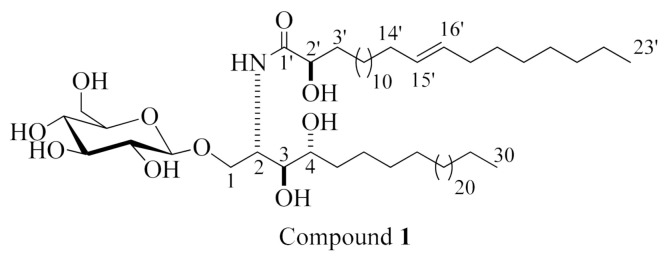
Chemical structure of compound **1**, holospiniferoside.

Compound **1** (displayed in Figure 1) was obtained as an amorphous white substance. Its molecular formula is suggested to be C_59_H_115_NO_10_, requiring three degrees of unsaturation based on its high-resolution mass spectrometry (HRMS) spectrum, which displayed a pseudo molecular-ion peak at *m/z* 1020.8417 [M + Na]^+^ (Supplementary Materials, Figure S1), and ^1^H and ^13^C NMR analysis. The ^1^H and ^13^C NMR spectral data of compound **1** are represented in Table 1 (Supplementary Materials, Figures S2–S6). The IR spectrum of this compound exhibited the characteristic absorption bands at λ 3244.7, 1613, and 1537 (amide functional group); 1159 (C–O); and 2922.8 and 721 (olefinic) cm^−1^ (Supplementary Materials, Figure S12) [20] The ^1^H NMR spectrum (measured in C_5_D_5_N, 400 MHz) of **1** indicated typical signals of an aliphatic chain, overlapped methyls at *δ*_H_ 0.85, and long methylene chain protons at *δ*_H_ 1.29, besides an amide group exchangeable protons at *δ*_H_ 8.58 (1H, d, *J* = 12.0 Hz), showing a sphingolipid backbone. Additionally, several characteristic resonances were noticed at *δ*_H_ 5.27 (1H, br m, H-2), 4.20 (1H, m, H-4), 4.35 (1H, m, H-3), 4.34, 4.54 (2H, m, H-1), and 4.74 (1H, t, 8.0, H-2’) as well as a signal for an anomeric proton at *δ*_H_ 4.96 (1H, d, *J* = 12 Hz). The ^13^C NMR spectrum (C_5_D_5_N, 100 MHz) displayed resonances for an amide carbonyl at *δ*_C_ 175.7 and two terminal methyl groups in aliphatic hydrocarbon chains at *δ*_C_ 14.1, and it exhibited characteristic signals at *δ*_C_ 51.6 (C-2), 70.4 (C-1), 72.3 (C-4), 72.4 (C-2’), and 75.7 (C-3) corresponding to a 2-amino-1,3,4,2’-tetrol part of the compound. Besides, an anomeric carbon at *δ*_C_ 105.5, in addition to other downfield-shifted resonances at *δ*_C_ 75.0, 78.3, 71.3, 78.4, and 62.5, was present, indicating a sugar part. The configuration of the glucopyranosyl moiety was determined to be *β*-configured according to the noticed coupling constant of the anomeric proton at *δ*_H_ 4.96 (1H, d, *J* = 12.0 Hz, H-1’’), indicating the diaxial coupling between H-1′’ and H-2′’ as well as the chemical shift of the anomeric carbon *δ*_C_ 105.5 ppm (in *α*-isomer, the coupling constant is 3.7 Hz and the chemical shift of the anomeric carbon would be at *δ*_C_ 98.5 ppm) [21,22]. The ^1^H NMR and ^13^C NMR spectral data attributed to a phytosphingosine-type cerebroside. For the determination of the length of the fatty acid chain, compound **1** was put through methanolysis, and peak detection by HRMS was then achieved following the method described by Sun et al. [23]. Briefly, compound **1** reacted with aqueous HCl/MeOH for methanolysis to obtain hydroxy acid methyl esters, phytosphingosine, and a sugar. The HRMS spectrum of the *α-*hydroxy fatty acid methyl ester of **1** (Supplementary Materials, Figure S11) displayed a molecular ion peak at *m/z* 383.3547 [M^+^] ascribed to a C_24_H_46_O_3_ fatty acid methyl ester, methyl (*E*)-2-hydroxytricos-15-enoate. To put an emphasis on the geometry of the double bond in the *α*-hydroxy fatty acid methyl ester (*m/z* 383.3547 [M^+^]), it was subjected to gas chromatography–mass spectrometry (GC–MS) analysis after oxidative cleavage by KMnO_4_ as described in [24], resulting in fatty acid methyl ester C_23_H_44_O_2_ with *m/z* 352. An analysis by GC–MS obtained fragments that confirmed the location of the double bond through the existence of masses at *m/z* 267, attributed to a [C_17_H_31_O_2_^•^] fragment; *m/z* 253, corresponding to [C_16_H_29_O_2_^•^]; and *m/z* 125, ascribed to [C_9_H_17_^•^] (Supplementary Materials, Figure S12). Hence, the placement of the double bond in 15’ was confirmed at methyl (*E*)-2-hydroxytricos-15-enoate. To identify the sugar moiety in compound **1**, the resulting sugar derivative from methanolysis was scanned by HPLC, and its retention time (t_R_) was compared with those of standard sugars. The obtained results revealed that the unknown sugar was glucose (t_R_ = 4.62 min). Additionally, the acetylated thiazolidine derivative of the obtained glucose from methanolysis was inspected by reversed-phase HPLC, and it exhibited the exact t_R_ of the acetylated thiazolidine analogue of standard D-glucose (19.7 min). The configuration of the cerebroside moiety was achieved via comparison of its ^1^H NMR, ^13^C NMR (measured in C_5_D_5_N), and physical data with those of the analogues reported previously, in which the chemical shifts of C-2 (*δ*_C_ 51.6), C-3 (*δ*_C_ 75.7), C-4 (*δ*_C_ 72.3), and C-2’ (*δ*_C_ 72.4), together with the chemical shifts of their corresponding protons besides the optical rotation +17.40 (*c* = 1.00, MeOH), were in congruence with those of the known reported cerebroside SJC-3 [23] and HPC-3 [25]. Thus, the configuration at C-2, C-3, C-4, and C-2’ was 2*S*, 3*S*, 4*R*, and 2’*R*, respectively. From the abovementioned data, the structure of compound **1** was elucidated as shown in Figure 1. As far as we know, it is a new compound, henceforth named holospiniferoside.

**Figure 2:**
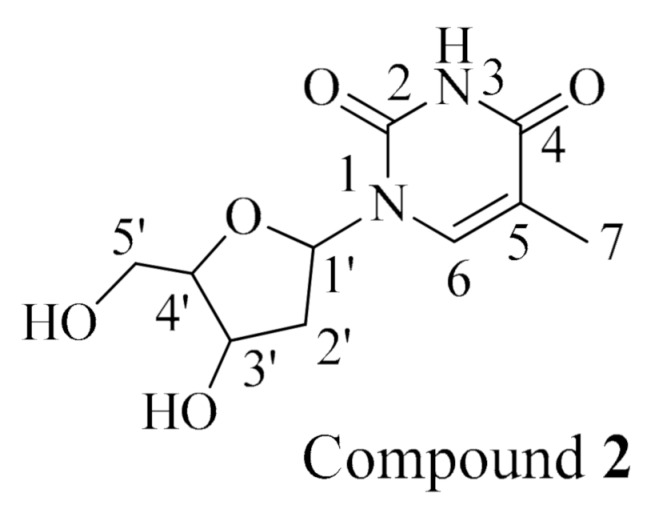
Chemical structure of compound **2**, thymidine.

Compound **2** (Figure 2) was identified as thymidine by comparing the ^1^H NMR and ^13^C NMR data with those reported in the literature [26] (Supplementary Materials, Figures S13 and S14). To the best of our knowledge, this is the first report of thymidine from *Holothuria spinifera*.

**Figure 3:**
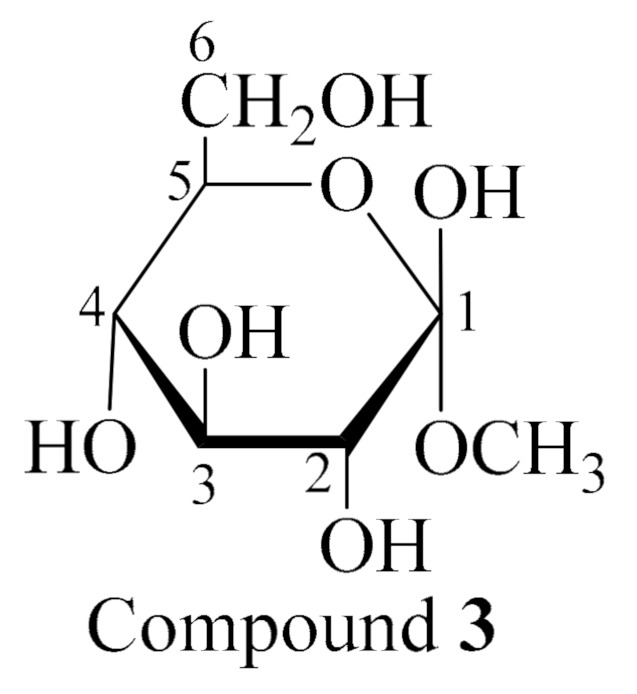
Chemical structure of compound **3**, methyl-*α*-d-glucopyranoside.

Compound **3** (Figure 3) was identified as methyl-*α*-d-glucopyranoside by comparing the ^1^H NMR and ^13^C NMR data with those reported in the literature [27] (Supplementary Materials, Figures S15 and S16). To the best of our knowledge, this is the first report of methyl-α-d-glucopyranoside from *Holothuria spinifera*.

**Figure 4:**
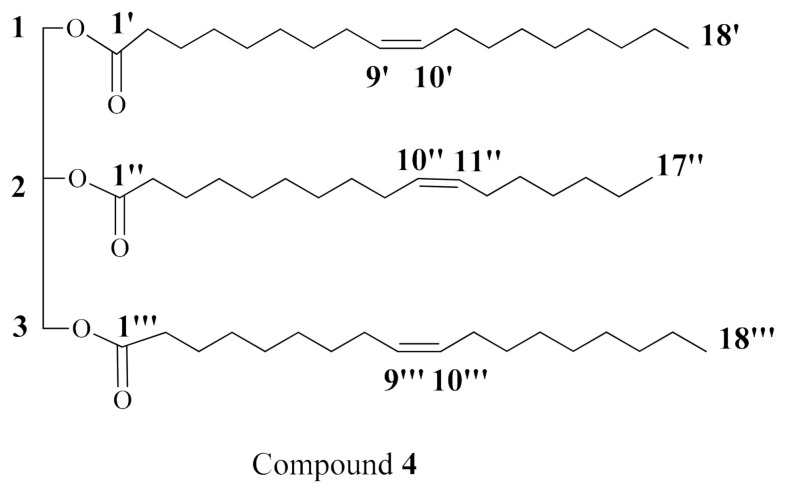
Chemical structure of compound **4**, triacylglycerol.

Compound **4** (Figure 4) was isolated as a white waxy substance. Its HRMS mass spectrum displayed a molecular ion peak at *m/z* 871.7609 [M + H]^+^ (Supplementary Materials, Figure S17). Its molecular formula was deduced to be C_56_H_102_O_6_, representing six degrees of unsaturation based on its NMR analysis. In addition, the IR spectrum of compound **4** displayed the characteristic absorption band at λ 1743.4 and 1159 Cm^−1^ for (C=O) and (C–O–C), respectively [28] (Supplementary Materials, Figure S18). The ^1^H and ^13^C NMR spectral data of compound **4** are listed in Table 2 (Supplementary Materials, Figures S19–S26). The interpretation of its NMR spectral data revealed the presence of two carbonyl signals at *δ*_C_ 173.2 (C-1’ and C-1’’’) and *δ*_C_ 172.8 (C-1’’), indicating three ester moieties along with the characteristic resonances of a glycerol backbone at *δ*_C_ 62.1 (C-1 and C-3) and *δ*_C_ 68.9 (C-2) together with their corresponding protons in the ^1^H NMR spectrum at *δ*_H_ 4.15 (2H, dd, *J* = 6, 12, H-1_a_ and H-3_a_), 4.29 (2H, dd, *J* = 6, 12, H-1_b_ and H-3_b_), and 5.25 (H-2). All these findings indicated the presence of a triacylglycerol [29]. Moreover, the presence of long alkyl chains was established by their characteristic chemical shifts in both ^13^C and ^1^H NMR spectra. In the ^1^H NMR spectrum, typical resonances of overlapped methyls at *δ*_H_ 0.88 and protons of methylene at *δ*_H_ 1.27 were present, while the attached proton test (APT) spectrum exhibited signals for terminal methyl at *δ*_C_ 14.0 and multiple signals for methylene carbons at the region from *δ*_C_ 22.7 to 34.2. The unsaturation of the fatty acids was deduced from the presence of olefinic carbons at *δ*_C_ 129.6 and 129.9 in the APT spectrum and their corresponding proton resonances at *δ*_H_ 5.26 and 5.35 in ^1^H NMR. For the determination of the fatty acid moieties, compound **4** was subjected to alkaline hydrolysis followed by methylation of the liberated fatty acids to obtain fatty acid methyl esters (FAME). The FAME were analyzed by GC–MS [30] (Supplementary Materials, Figures S27 and S28), which afforded (*Z*)-10-heptadecenoic acid methyl ester C_18_H_34_O_2_ (*m*/*z* 282 [M]^+^) and (*Z*)-9-octadecenoic acid methyl ester (oleic acid methyl ester) C_19_H_36_O_2_ (*m*/*z* 296 [M]^+^). The geometry of the double bonds was further confirmed by the chemical shifts of the allylic carbons at *δ*_C_ 27.1 and 27.2, which characterize the (*Z*)-configuration of the double bond [31]. The two oleate moieties were suggested to be in positions 1 and 3 based on the presence of only two carbonyl carbons for three ester moieties, indicating the symmetry of the structure, also making an achiral compound. From the above data, the structure of compound **4** was assigned as a 1,2,3-*O*,*O*,*O*-triacyl derivative of glycerol esterified with two molecules of (*Z*)-9-octadecenoic acid and (*Z*)-10-heptadecenoic acid. To the best of our knowledge, this is a new compound, 1,2,3-*O*,*O*,*O*-triacyl derivative of glycerol from *Holothuria spinifera*.

**Figure 5:**
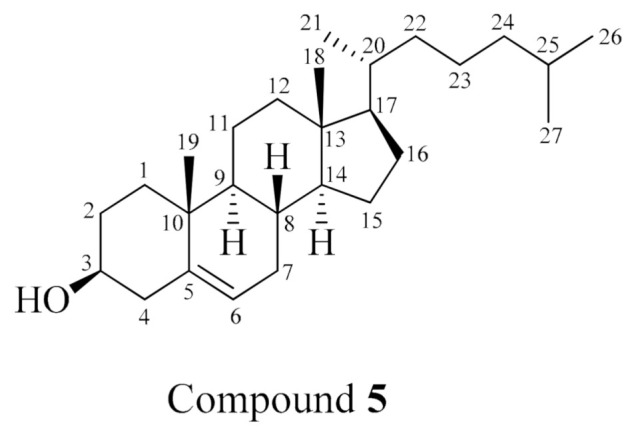
Chemical structure of compound **5**, cholesterol.

Compound **5** (Figure 5) was identified as cholesterol by comparing its NMR data with the literature [32] (Supplementary Materials, Figures S29–S31). To the best of our knowledge, this is the first report of cholesterol from *Holothuria spinifera*.

### 2.2. Evaluation of the Antitumor Activity of Holospiniferoside (**1**) In Vitro

The cytotoxicity and the antiapoptotic effects of ceramides and cerebrosides against a wide array of cancer cell lines have been reported in several studies [12,33,34,35]. In addition, the crude extract of *H. spinifera* has been reported to possess potent cytotoxic potential against MCF7 with an IC_50_ value of 4.58 µg/mL [12]. Therefore, the cytotoxicity of the new cerebroside (compound **1)** was assessed on breast cancer cell line (MCF-7) by the sulforhodamine B (SRB) assay using the method described in [12,36,37]. The color intensity was measured, and the IC_50_ values were then calculated using an ELISA reader. As listed in Table 3, compound **1** showed promising anticancer activity against the breast cancer (MCF-7) cell line with an IC_50_ value of 20.6 µM, which is similar to that of cisplatin (15.3 µM) as the standard drug.

### 2.3. Molecular Docking Studies

**Figure 6:**
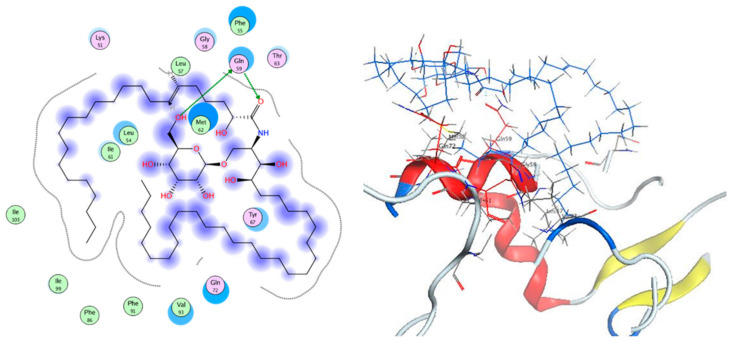
2D and 3D captions of compound **1**.

In cancer, p53 is a tumor suppressor gene that can be inhibited by overexpressed Mdm2. Based on the fact that Mdm2 is a ubiquitin ligase, this may result in p53 ubiquitination and consequently p53 proteasomal degradation [38]. Therefore, one of the strategies of the anticancer design is the inhibition of the Mdm2–p53 interaction using different inhibitors. Several compounds such as cerebrosides, triacylglycerols, and steroids have proven to exert apoptotic activity [39,40]. To understand the antitumor activity of these compounds, docking studies were carried out on the crystal structures of Mdm2 and 3LBK [41]. Analysis of the results revealed that the new cerebroside, compound **1**, showed two hydrogen bond interactions via its alcoholic hydroxy group and its carbonyl group with the Gln 59 amino acid residue, showing a binding affinity score of −12.133 kcal/mol (Figure 6). This result is consistent with the significant inhibitory activity of the mentioned cerebroside against the breast cancer cell line, displaying an IC_50_ value of 20.6 µM.

## 3. Experimental Section

### 3.1. General Experimental Procedures

^1^H NMR (400 MHz) and ^13^C NMR (100 MHz) spectral data were obtained on a Varian AS 400 (Varian Inc., Palo Alto, CA, USA) machine applying the residual solvent signal as an internal standard. Bruker BioApex (Bruker Corporation, Bruker Optics, Karlsruhe, Germany) instrument was utilized for recording the high-resolution mass spectra. IR. Spectra were recorded using FT-IR spectrometer (Alpha II, Bruker Optics, Germany) For chromatographic separations, silica gel (Purasil 60 Å, 230–400 mesh) (Whatman, Sanford, ME, USA), pre-coated silica gel G-25 UV254 TLC plates (20 cm × 20 cm) (E. Merck, Darmstadt, Germany), and Sephadex LH-20 (Sigma Aldrich^®^, Darmstadt, Germany) were used.

### 3.2. Sea Cucumber Material

Sea cucumber *Holothuria spinifera* material was obtained from Sharm El Sheikh in the Egyptian Red Sea. The collected material was air-dried and then stored at −24 °C until further processing. A voucher specimen (Registration # SAA-129) was placed at the Herbarium Section of the Pharmacognosy Department, Faculty of Pharmacy, Suez Canal University, Ismailia, Egypt.

### 3.3. Extraction and Isolation

In our previous work, 2 kg of sea cucumber *Holothuria spinifera* was frozen and cut into small pieces and then extracted with a mixture of MeOH/CH_2_Cl_2_ (1:1) until exhaustion at room temperature. The combined extracts were dried under reduced pressure to afford 100 g of crude extract. The obtained extract was fractionated by VLC. Gradient elution was applied commencing with *n*-hexane. The polarity was then increased by EtOAc and MeOH to afford nine fractions: HS-1 to HS-9. Fraction HS-5 (EtOAc/MeOH 75:25) (2.52 g) was rechromatographed on a silica gel column using 100% CHCl_3_ as an eluent followed by gradients CHCl_3_/MeOH (65:35) to obtain eight subfractions (HS-5-1 to HS-5-8). Repeated chromatographic separation of HS-4, HS-5-3, HS-5-4, and HS-5-6 resulted in the isolation of a ceramide molecular species and two cerebrosides, in addition to cholesterol sulfate [12].

On continuation of the aforementioned phytochemical investigation of *H. spinifera*, subfraction HS-5-1 (170 mg) was subjected to silica gel column chromatography applying gradients CHCl_3_/MeOH commencing with 90:10 and culminating with 65:35. Then, final purification was achieved by rechromatography using Sephadex. LH-20 column eluted with CHCl_3_/MeOH (1:1) to afford compound **1** (20 mg, white amorphous powder). The subfraction HS-S-8 (290 mg) was rechromatographed on a silica gel column eluted with CHCl_3_/MeOH (9:1 to 6.5:3.5). A pure solid material was obtained (compound **2**, 10 mg) and a subfraction HS-S-8-1 (50 mg), which was further purified by Sephadex LH-20 using CHCl_3_/MeOH (1:1) to give a white pure solid substance, compound **3** (12 mg, Rf = 0.37; TLC system: 10% MeOH in CHCl_3_).

Fraction HS-2 (25:75 EtOAc/*n*-hexane) (1.87 g) was placed on a silica gel column chromatography and eluted initially with 100% *n*-hexane followed by gradient systems of *n*-hexane, EtOAc and MeOH till 50% EtOAc in MeOH. Ten subfractions (HS-2a to HS-2i) were obtained. Subfraction HS-2g (480 mg) was slurred with a small amount of silica, placed on the top of the silica gel column, and eluted with 5% EtOAc in *n*-hexane with a gradient increase of EtOAc until reaching 100% EtOAc. Compound **4** (65 mg) was obtained in 25% EtOAc in *n*-hexane and further purified with Sephadex LH-20. Fraction HS-3 (50% EtOAc in *n*-hexane + 75% EtOAc in *n*-hexane) (0.75 g) was placed on a silica gel column, and elution was then performed initially with 10% EtOAc in *n*-hexane followed by increasing the polarity in a gradient mode until reaching 50% MeOH in EtOAc. A pure white substance was obtained, compound **5** (65 mg), which was then further purified with Sephadex LH-20.

### 3.4. Cerebroside Hydrolysis

Compound **1** (2 mg) was hydrolyzed and methylated according to the method described in [12,33] in order to identify the fatty acid moiety in the compound. The prepared FAME was subjected to HRMS analysis. For determination of the double bond position in the fatty acid part in compound **1**, the prepared *α-*hydroxy fatty acid methyl esters was subjected to oxidative cleavage via Lemieux oxidation by applying the method described in Sun et al. [23]. Then, the resulting fatty acid methyl ester was used for GC–MS analysis. The determination of FAME was simplified utilizing the AMDIS software (www.amdis.net) using their retention indices for identification (mass spectrum matching to authentic standards, Wiley spectral library collection database).

### 3.5. Identification of the Sugar Moiety in Compound ***1***

The identification of the sugar moiety of compound **1** was achieved by the procedure mentioned in [12,33,42], where the methylated sugar derivative was prepared followed by HPLC analysis (COSMOSIL Sugar-D, 4.6 ID × 250 mm, 1 mL/min, 95% acetonitrile) utilizing a RI detector. The obtained *t_R_* of the sugar analogue was compared with those of standard glucose (t_R_ at 4.62) and galactose (t_R_ at 4.76).

### 3.6. Determination of the Configuration of the Sugar Moiety in ***1***

The absolute configuration of the sugar part of compound **1** was determined by applying the method described in [12,33,42], where the acetylated thiazolidine derivative of the liberated sugar was prepared and then analyzed by reversed-phase HPLC (Cosmosil-5C_18_-AR-II, 4.6ID × 250 mm, 0.8 mL/min, UV detector (250 nm, 25% acetonitrile in 50 mM H_3_PO_4_). The *t_R_* of the prepared derivative was compared with those of standards.

### 3.7. Hydrolysis of Triacylglycerol ***4***

After methanolysis, GC–MS analysis of the prepared FAME of compound **4** was performed, where compound **4** was dissolved in benzene and refluxed with 10% ethanolic KOH for 24 h. The reaction mixture was extracted with ether after dilution with water. The saponifiable part was acidified with concentrated HCl, and liberated fatty acids were extracted with ether and then concentrated to yield three fatty acids. The prepared fatty acids of compound **4** were reacted with MeOH in the presence of concentrated H_2_SO_4_ for 1 h. After dilution of the reaction mixture with water, the obtained fatty acid methyl esters were extracted with ether, dried with anhydrous magnesium sulfate, concentrated, and analyzed by GC–MS.

### 3.8. Spectroscopic Data

#### 3.8.1. Holospiniferoside (**1**)

White powder; HRMS: *m*/*z* 1020.8417 [M + Na]^+^; ^1^H NMR (C_5_D_5_N, 400 MHz) and ^13^C NMR (C_5_D_5_N, 100 MHz) spectral data, see Table 1.

#### 3.8.2. Thymidine (**2**)

White amorphous powder; ^1^H NMR (C_5_D_5_N, 400 MHz): δ_H_ 8.14 (1H, s, NH), 7.03 (1H, t, *J* = 8 Hz, H-1’), 4.98 (2H, H-3’, brs), 4.46 (1H, q, *J* = 3.0 Hz, H-4’), 4.24 (1H, dd, *J* = 16, 4 Hz, H-5’a), 4.15 (1H, dd, *J* = 16, 4 Hz, H-5’b), 2.66–2.60 (2H, m, H-2’), 1.85 (3H, s, Me); ^13^C NMR (C_5_D_5_N, 100 MHz): δ_C_ 164.9 (C-4), 151.9 (C-2), 136.6 (C-6), 110.4 (C-5), 88.8 (C-4’), 85.2 (C-1’), 71.4 (C-3’), 62.2 (C-5’), 41.3 (C-2’), 12.7 (Me, C-7).

#### 3.8.3. Methyl-α-d-glucopyranoside (**3**)

White amorphous powder; ^1^H NMR (C_5_D_5_N, 400 MHz): *δ*_H_ 5.15 (1H,d, *J* = 6 Hz, H-1), 4.24 (1H, m, H-3), 4.13 (1H, dd, *J* = 8,4 Hz, H-2), 4.21(1H, br,s, H-4), 3.51(1H, m, H-5), 4.36 (1H, m, H6_a_), 4.50 (1H, m, H6*_b_*), 3.44 (1H, s, OCH_3_); *δ*c 101.4 (C-1), 73.8 (C-2), 75.4 (C-3), 72.1 (C-4), 74.1 (C-5), 62.8 (C-6), 54.8 (OCH_3_).

#### 3.8.4. Triacylglycerol (**4**)

White powder; HRMS: *m*/*z* 871.7609 [M + H]^+^. ^1^H NMR (CDCl_3_, 400 MHz) and ^13^C NMR (CDCl_3_, 100 MHz) spectral data, see Table 2.

#### 3.8.5. Cholesterol (**5**)

White amorphous powder; ^1^H NMR (CDCl_3_, 400 MHz): *δ*_H_ 5.36 (1H, m, H-6), 3.53 (1H, m, H-3), 2.27 (2H, m, H-4), 2.00 (2H, m, H-7), 1.86 (2H, m, H-1), 1.50 (2H, m, H-2), 1.15–2.33 (12H, m, H-8, H-9, H-12, H-20, H-22, H-23, H-24, H-25), 1.02 (3H, s, H-19), 0.93 (2H, m, H-11), 0.88 (6H, m, H-26, H-27), 0.85 (3H, m, H-21), 0.70 (3H, s, H-18); ^13^C NMR (CDCl_3_, 100 MHz): *δ*_C_ 140.8 (C-5), 121.7 (C-6), 71.8 (C-3), 50.1 (C-9), 42.3 (C-4), 39.8 (C-12), 39.8 (C-24), 37.2 (C-1), 36.5 (C-10), 36.2 (C-20), 33.9 (C-22), 31.6 (C-2), 31.6 (C-7), 31.6 (C-8), 28.9 (C-25), 23.9 (C-23), 21.1 (C-11), 19.6 (C-27), 19.4 (C-26), 19.0 (C-19), 18.8 (C-21), 11.7 (C-18).

### 3.9. Cytotoxicity Assays

The cytotoxicity of compound **1** on MCF-7 breast cancer cell line was evaluated by the SRB assay according to the method previously mentioned in Abdelhameed et al. and Skehan et al. [13,35] using an ELISA microplate reader (Sunrise Tecan reader, Germany). The optical density (O.D.) of each well was estimated spectrophotometrically at λ 570 nm. The experiment was performed thrice, and the IC_50_ values of the test compounds were calculated.

### 3.10. Molecular Docking Studies

In the Protein DataBank, the crystallographic structures of 3LBK in complex with its ligand is accessible [40]. Structure arrangement process was applied to inspect the protein errors, and the creation of a reasonable protein structure was established on default rules on the Molecular Operating Environment 2019.0101 software (MOE). The Gasteiger methodology was finally utilized to estimate the partial charges of the protein. Molecular docking simulations were carried out with MOE of Chemical Computing Group Inc. on Core i5 2.2 GHz workstation operating on a Windows 10 PC [43]. Compound **1** coordinate was established applying ChemDraw Ultra 11.0. After that, its protonation, correction of atoms, and bond types were clearly settled; hydrogen atoms were placed and protonation and finally minimization was carried out (AMBER10, gradient: 0.01). The docking investigation was confirmed by redocking the ligand in PDB structure 3LBK, and the ligand was then removed. The default Triangle Matcher placement procedure was chosen for molecular simulation. GBVI/WSA dG scoring function, which estimates the free energy of binding of the ligand from a given pose, was selected to order the final poses. The ligand complex with the protein possessing the lowest S-score was picked out. The redocking of the ligand with its target exhibited a RMSD value of 0.806 Å, which verifies that the ligand binds to the same pocket and confirms the dependability of docking parameters.

## 4. Conclusions

In continuation of our research toward the isolation of bioactive metabolites of marine origin, we herein report that the phytochemical investigation of the sea cucumber *H. spinifra* resulted in the isolation of the new cerebroside holospiniferoside (**1**), a new triacylglycerol, and three known compounds **2**, **3,** and **5**. All the isolated compounds are reported in the Red Sea cucumber *Holothuria spinifera* for the first time. Holospiniferoside (**1**) exhibited promising cytotoxic potential against MCF-7 cancer cells (IC_50_ = 20.6 µM). Moreover, it displayed strong interaction with the active site of the Mdm2–p53 protein, suggesting its apoptotic activity. In brief, marine organisms could be considered as gold mines of promising anticancer drugs.

## Figures and Tables

**Table 1:** ^1^H (400 MHz) and ^13^C NMR (100 MHz) data for the new compound **1** in C_5_D_5_N.

1		
Position	*δ_H_* (mult., *J*_Hz_)	*δ_C_*
1	4.34 (H1_a_, m) 4.54 (H1_b_, m)	70.4
2	5.27 (1H, br, m)	51.6
3	4.34 (1H, m)	75.7
4	4.20 (1H, m)	72.3
5	1.68 (2H, m)	34.0
6	1.29 *	29.4
7	1.29 *	30.0
8	1.29 *	30.0
9–29	1.29 *	29.8
30	0.85 (3H, t, 8)	14.1
1’	-	175.7
2’	4.74 (1H, m)	72.4
3’	1.87 (1H, m)	34.0
4’–13’	1.23 (2H, m)	29.4
14’	2.08 (2H, m)	32.0
15’	5.27 (1H, m)	130.1
16’	5.49 (1H, m)	130.1
17’	2.19 (2H, m)	32.9
18’–22’	1.23 (2H, m)	29.4
23’	0.85 (3H, t, 8)	14.1
NH	8.58 (1H, d, 12)	-
1’’	4.96(1H, d, 12)	105.5
2’’	4.02(1H, m)	75.0
3’’	4.54 (1H, m)	78.3
4’’	4.74 (1H, m)	71.3
5’’	3.85 (1H, br, m)	78.4
6’’	4.35 (1H, dd, 4.0, 8.0) 4.53 (1H, m)	62.5

* Overlapped signals are listed without multiplicity.

**Table 2:** ^1^H (400 MHz) and ^13^C NMR (100 MHz) data for compound **4** in CDCl_3_.

			4		
Position	*δ_H_* (mult., *J*_Hz_)	*δ_C_*	Position	*δ_H_* (mult., *J*_Hz_)	*δ_C_*
1	4.14 (H_a_,dd, 12,6) 4.29 (H_b_,dd, 12,6)	62.1	9’’	2.01 (2H, m)	27.2–27.4 *
2	5.25 (1H, m)	68.9	10’’	5.33 (1H, m)	129.6–129.9 *
3	4.14 (H_a_,dd, 12,6) 4.29 (H_b_,dd, 12,6)	62.1	11’’	5.33 (1H, m)	129.6–129.9 *
1’	-	173.2	12’’	2.01 (2H, m)	27.2–27.4
2’	2.30 (2H, m)	34.0–34.2 *	13’’–15’’	1.27 (6H, m)	29.0–29.8 *
3’	1.60 (2H, m)	24.8–24.9 *	16’’	1.27 (2H, m)	22.7
4’–7’	1.27 (8H, m)	29.0–29.8 *	17’’	0.88 (3H, m)	14.1
8’	2.01 (2H, m)	27.2–27.4 *	1’’’	-	173.1
9’	5.33 (1H, m)	129.6–129.9 *	2’’’	2.30 (2H, m)	34.0–34.2 *
10’	5.33 (1H, m)	129.6–129.9 *	3’’’	1.60 (2H, m)	24.8–24.9 *
11’	2.01 (2H, m)	27.2–27.4 *	4’’’’–7’’’	1.27 (8H, m)	29.0–29.8 *
12’–15’	1.27 (8H, m)	29.0–29.8 *	8’’’	2.01 (2H, m)	27.2–27.4 *
16’	1.27 (2H, m)	31.8–31.9 *	9’’’	5.33 (1H, m)	129.6–129.9 *
17’	1.27 (2H, m)	22.7	10’’’	5.33 (1H, m)	129.6–129.9 *
18’	0.88 (3H, m)	14.1	11’’’	2.01 (2H, m)	27.2–27.4 *
1’’	-	172.8	12’’–15’’’	1.27 (8H, m)	29.0–29.8 *
2’’	2.30 (2H, m)	34.0–34.2 *	16’’’	1.27 (2H, m)	31.8–31.9 *
3’’	1.60 (2H, m)	24.8–24.9 *	17’’’	1.27 (2H, m)	22.7
4’’–8’’	1.27 (10H, m)	29.0–29.8 *	18’’’	0.88 (3H, m)	14.1

dd means doublet of doublet; * Overlapped signals are listed without multiplicity.

**Table 3:** IC^50^ of compound **1** on the breast cancer cell line (MCF-7).

Sample.	IC_50_ (µM)	HFB-4 IC_50_ (µM)
Compound **1**	20.6 ± 0.03	>200
Cisplatin	15.3 ± 0.02	>200

Each data point represents the mean ± SD of four independent experiments (significant differences at *p* < 0.05).

## Data Availability

Data is contained within the article or supplementary material.

## Supplementary Materials

The following are available online, Figures S1–S10 LC-HRESIMS, ^1^H NMR, ^13^C NMR and IR compound **1**, Figures S11 and S12: HRMS & GC-MS of compound **1**, Figures S13 and S14: ^1^H NMR, ^13^C NMR of compound **2**, Figures S15 and S16 ^1^H NMR, ^13^C NMR of compound **3**, Figures S17–S28 LC-HRESIMS, ^1^H NMR, ^13^C NMR of compound **4**, Figures S29–S31: ^1^H NMR, ^13^C NMR of compound **5**.

## Sample Availability

Samples of the compounds are available from the authors.
