# Supplementary material for: Holospiniferoside: A New Antitumor Cerebroside from The Red Sea Cucumber Holothuria spinifera: In Vitro and In Silico Studies
Source: Molecules. 2021 Mar 12;26(6):1555. doi: 10.3390/molecules26061555 (PMC8001240; doi:10.3390/molecules26061555)
Supplement: Supplementary file 1 [file molecules-26-01555-s001.pdf]

molecules

MDPI

Article

Holospiniferoside: A New Antitumor Cerebroside from The Red Sea Cucumber *Holothuria spinifera*: In Vitro and In Silico Studies

Enas E. Eltamany [1,†], Usama Ramadan Abdelmohsen [2,3,†], Dina M. Hal [1], Amany K. Ibrahim [1], Hashim A. Hassanean [1], Reda F.A. Abdelhameed [1], Tarek A Temraz [4], Dina Hajjar [5], Arwa A. Makki [5], Omnia Magdy Hendawy [6,7], Asmaa M. AboulMagd [8], Khayrya A. Youssif [9], Gerhard Bringmann [10,*], Safwat A. Ahmed [1,*]

[1] Department of Pharmacognosy, Faculty of Pharmacy, Suez Canal University 41522 Ismailia, Egypt; enastamany@gmail.com (E.E.E.); dina_hal@pharm.suez.edu.eg (D.M.H.); am_kamal66@yahoo.com (A.K.I.); amahdali@gmail.com (H.A.H.); omarreda_70@yahoo.com (R.F.A.A.)
[2] Department of Pharmacognosy, Faculty of Pharmacy, Deraya University 61111 New Minia, Egypt; usama.ramadan@mu.edu.eg
[3] Department of Pharmacognosy, Faculty of Pharmacy, Minia University 61519 Minia, Egypt
[4] Department of Marine Science, Faculty of Science, Suez Canal University 41522 Ismailia, Egypt; ttemraz@science.suez.edu.eg
[5] Department of Biochemistry, Collage of Science, University of Jeddah 80203 Jeddah, Saudi Arabia; dhajjar@uj.edu.sa (D.H.); amaki@uj.edu.sa (A.A.M.)
[6] Department of Chemistry of Pharmacology, Faculty of Pharmacy, Jouf University, 2014, Saudi Arabia; omhendawy@ju.edu
[7] Department of clinical Pharmacology, Faculty of Medicine, Beni Suef University, 62513 Beni-Suef, Egypt
[8] Pharmaceutical Chemistry Department, Faculty of Pharmacy, Nahda University, 62513 Beni Suef, Egypt; asmaa.aboulmaged@nub.edu.eg
[9] Department of Pharmacognosy, Faculty of Pharmacy, Modern University for Technology and Information 12585 Cairo, Egypt; khayrya.youssif@gmail.com
[10] Institute of Organic Chemistry, University of Würzburg, Am Hubland, 97074 Würzburg, Germany
* Correspondence: bringman@chemie.uni-wuerzburg.de (G.B.); safwat_aa@yahoo.com (S.A.A.); +49-931-318 5323 (G.B.); +20-010-92638387 (S.A.A.); +49-931-318 4755 (G.B.); +20-064-3230741 (S.A.A.)
† These authors contributed equally to this work.

Figure S1: HRMS of Compound 1 $(M+Na)^+$. 4
Figure S2: ^{1}H-NMR spectrum of compound 1 in (C_5D_5N, 400 MHz). 5
Figure S3: Partial expansion of the ^{1}H-NMR spectrum of compound 1 in (C_5D_5N, 400 MHz). 6
Figure S4: Partial expansion of the ^{1}H-NMR spectrum of compound 1 in (C_5D_5N, 400 MHz). 7
Figure S5: Partial expansion of the ^{1}H-NMR spectrum of compound 1 in (C_5D_5N, 400 MHz). 8
Figure S6: ^{13}C-NMR spectrum of compound 1 in (C_5D_5N, 100 MHz). 9
Figure S7: Partial expansion of the ^{13}C-NMR spectrum of compound 1 in (C_5D_5N, 100 MHz). 10
Figure S8: Partial expansion of the ^{13}C-NMR spectrum of compound 1 in (C_5D_5N, 100 MHz). 11
Figure S9: Partial expansion of the ^{13}C-NMR spectrum of compound 1 in (C_5D_5N, 100 MHz). 12
Figure S10: IR spectrum of compound 1....13

Figure S11: HRMS of *α*-hydroxy fatty acid methyl ester resulted from hydrolysis of compound 1....14
Figure S12: GC-MS analysis of fatty acids methyl ester carried out after oxidation of *α*-hydroxy fatty acid methyl ester (Compound 1)....15
Figure S13: ^{1}H-NMR spectrum of compound 2 in (C_5D_5N, 400 MHz). 16
Figure S14: ^{13}C-NMR spectrum of compound 2 in (C_5D_5N, 100 MHz). 17
Figure S15: ^{1}H -NMR spectrum of Compound 3 in (C_5D_5N, 400 MHz). 18
Figure S16: ^{13}C-NMR spectrum of Compound 3 (C_5D_5N, 100 MHz). 19
Figure S17: HRMS of Compound 4 $(M+H)^+$....20
Figure S18: IR Compound 4 $(M+H)^+$....21

Figure S19: ^{1}H-NMR spectrum of compound 4 in ($CDCl_3$, 400 MHz). 22

Figure S20: Partial expansions of the ^{1}H-NMR spectrum of compound 4 in ($CDCl_3$, 400 MHz) 23
Figure S21: Partial expansions of the ^{1}H-NMR spectrum of compound 4 in ($CDCl_3$, 400 MHz) 24
FigureS 22: Partial expansions of the ^{1}H-NMR spectrum of compound 4 in ($CDCl_3$, 400 MHz) 25
Figure S23: ^{13}C-NMR spectrum of compound 4 in ($CDCl_3$, 400 MHz). 26
Figure S24: Partial expansion ^{13}C-NMR spectrum of compound 4 in ($CDCl_3$, 400 MHz). 27
Figure S25: Partial expansion ^{13}C-NMR spectrum of compound 4 in ($CDCl_3$, 400 MHz). 28
Figure S26: Partial expansion ^{13}C-NMR spectrum of compound 4 in ($CDCl_3$, 400 MHz). 29
Figure S27: MS spectrum for 10-Heptadecenoic acid (z)- methyl ester. 30
Figure S28: MS spectrum for 9-Octadecenoic acid (z)-methyl ester 31
Figure S29: ^{1}H -NMR spectrum of Compound 5 in ($CDCl_3$, 400 MHz). 32
Figure S30: ^{13}C-NMR spectrum of Compound 5 ($CDCl_3$, 100 MHz). 33
Figure S31: DEPT spectrum of Compound 5 in ($CDCl_3$, 100 MHz). 34

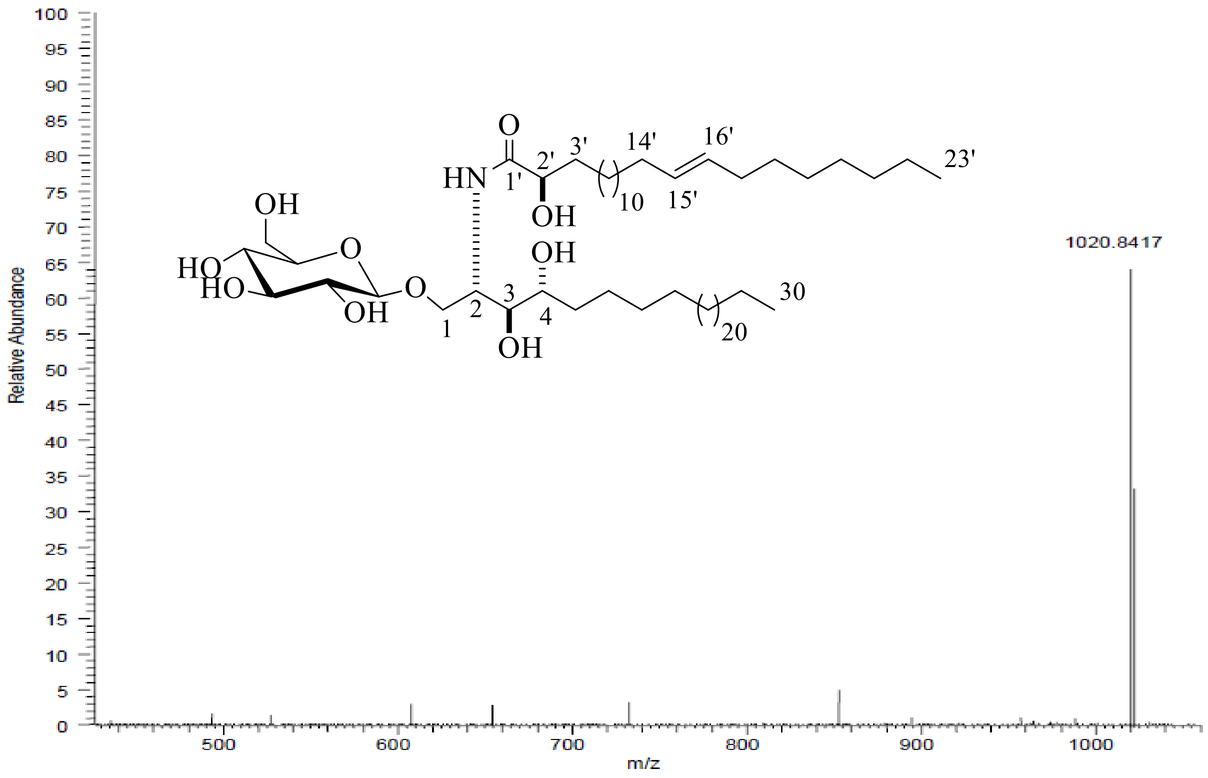

Figure S1: HRMS of Compound 1 $(M+Na)^+$.

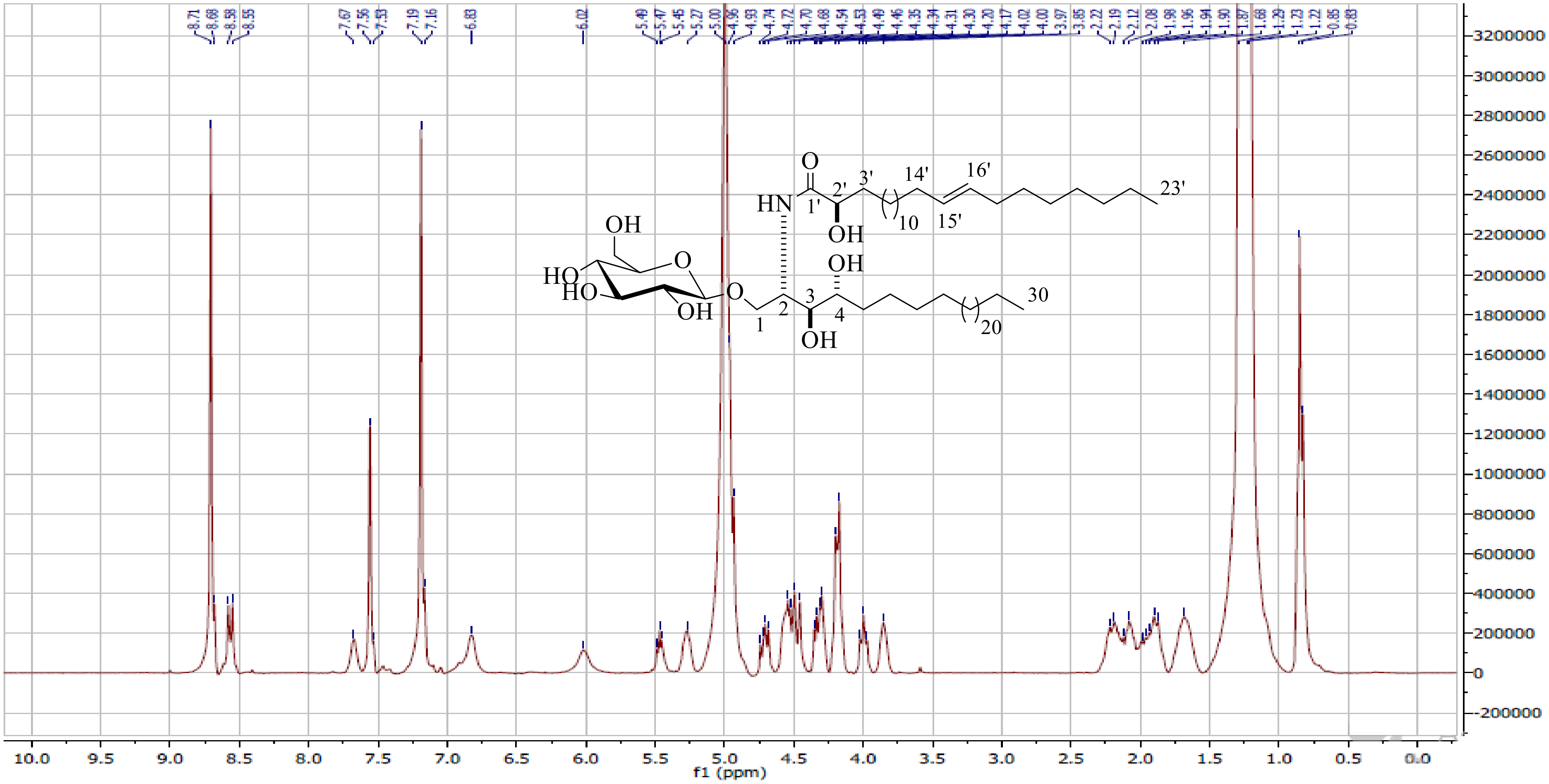

Figure S2: ^{1}H-NMR spectrum of compound 1 in (C_5D_5N, 400 MHz).

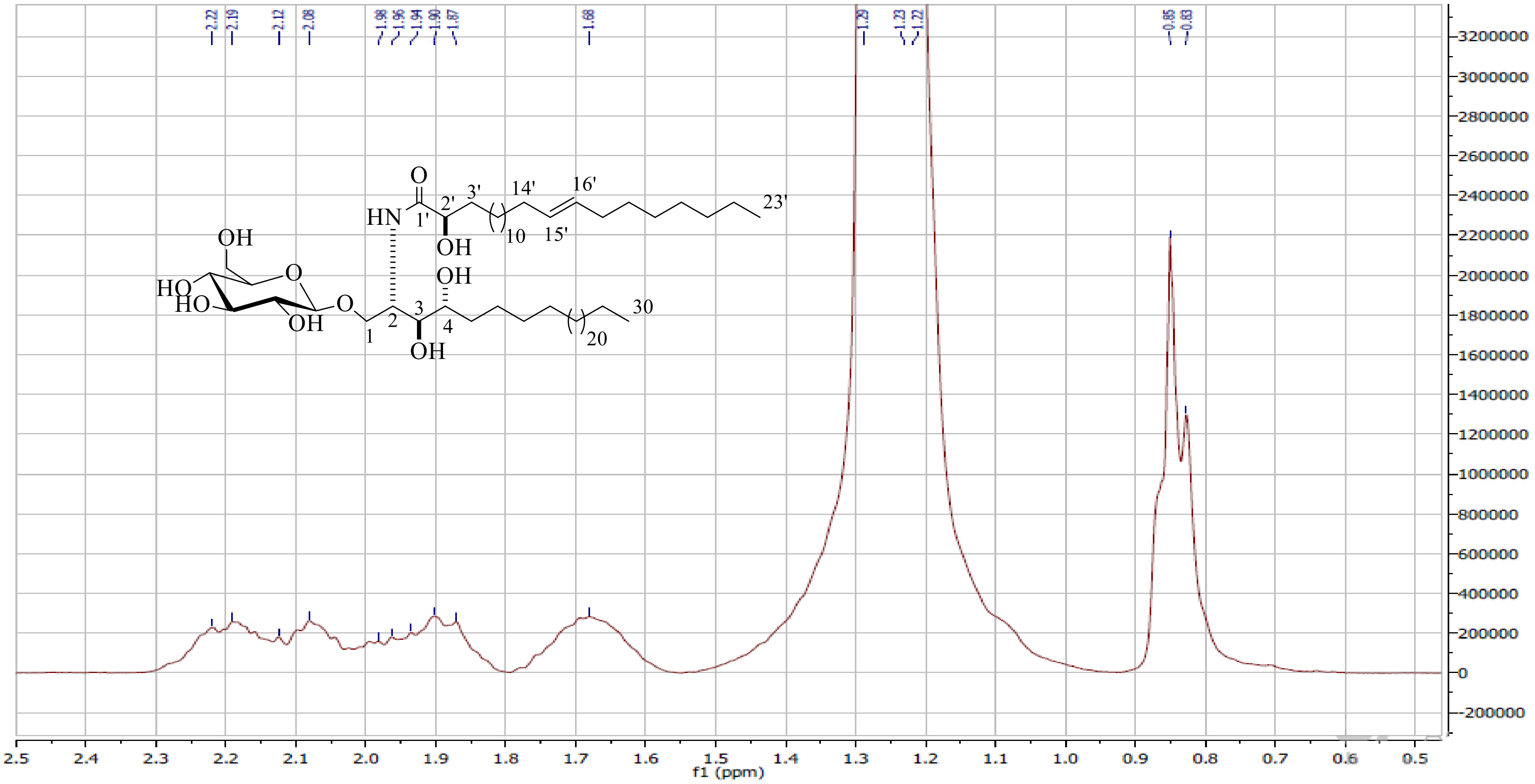

Figure S3: Partial expansion of the ^{1}H-NMR spectrum of compound 1 in (C_5D_5N, 400 MHz).

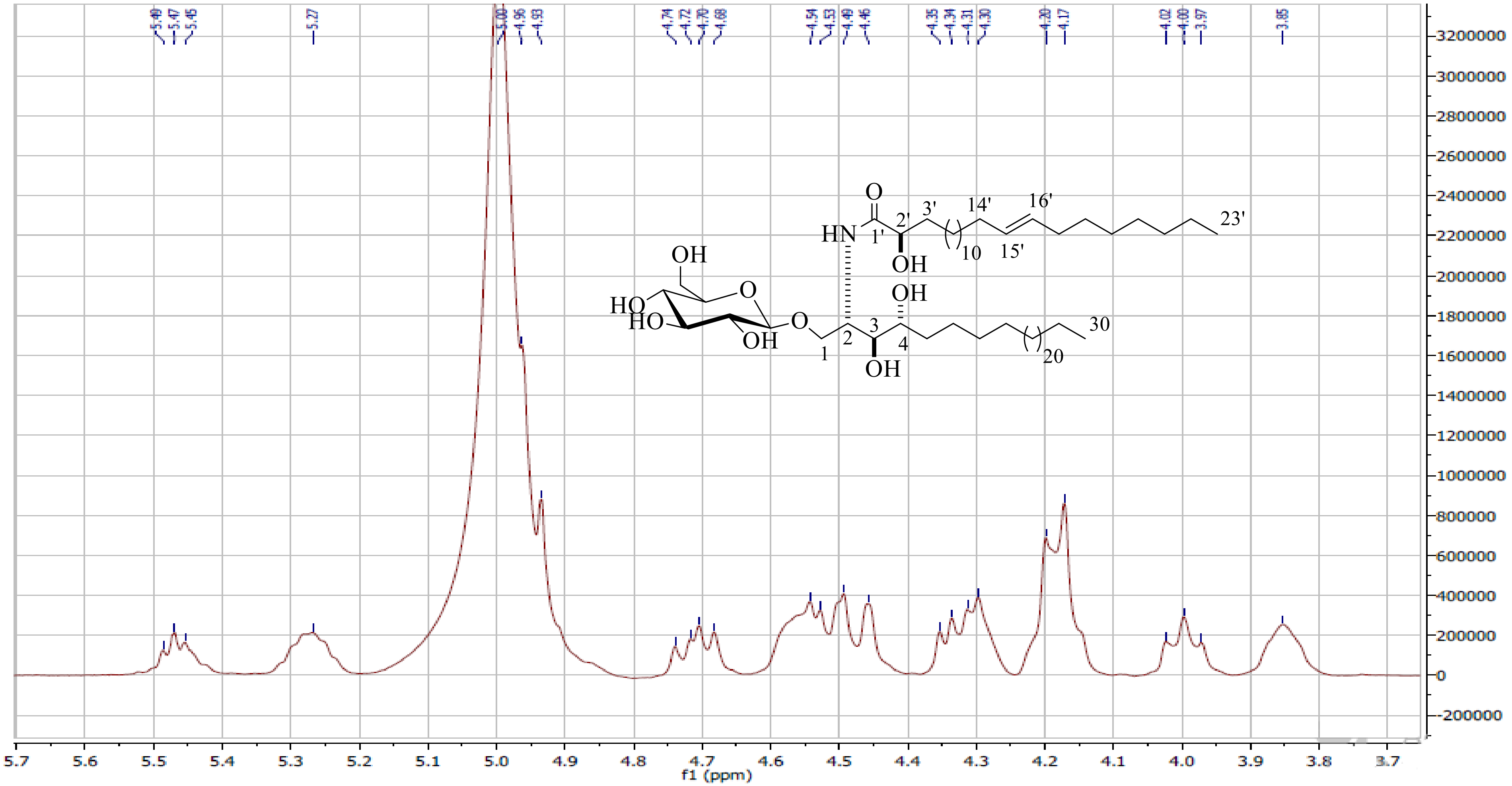

Figure S4: Partial expansion of the ^{1}H-NMR spectrum of compound 1 in (C_5D_5N, 400 MHz).

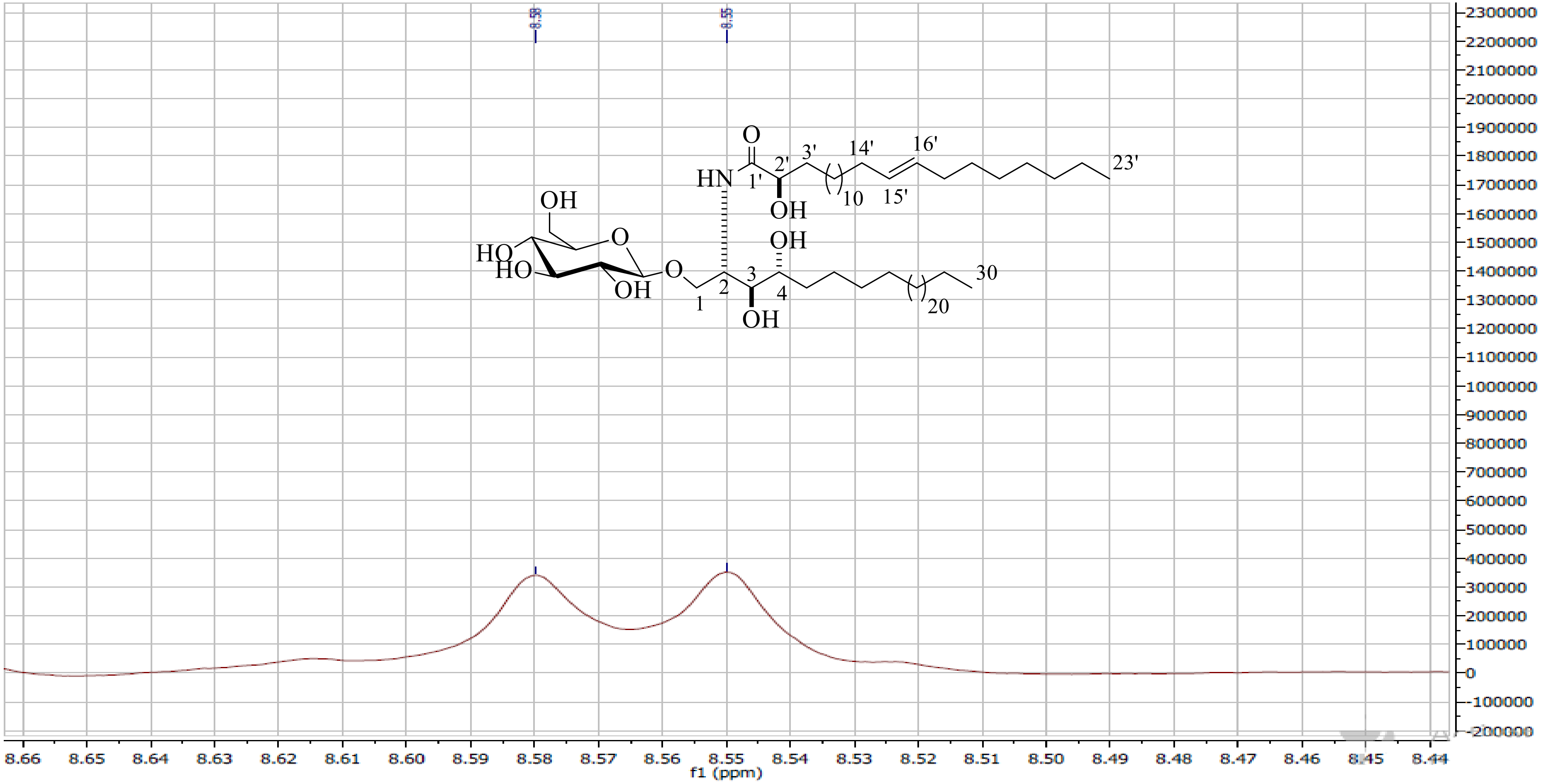

Figure S5: Partial expansion of the ^{1}H-NMR spectrum of compound 1 in (C_5D_5N, 400 MHz).

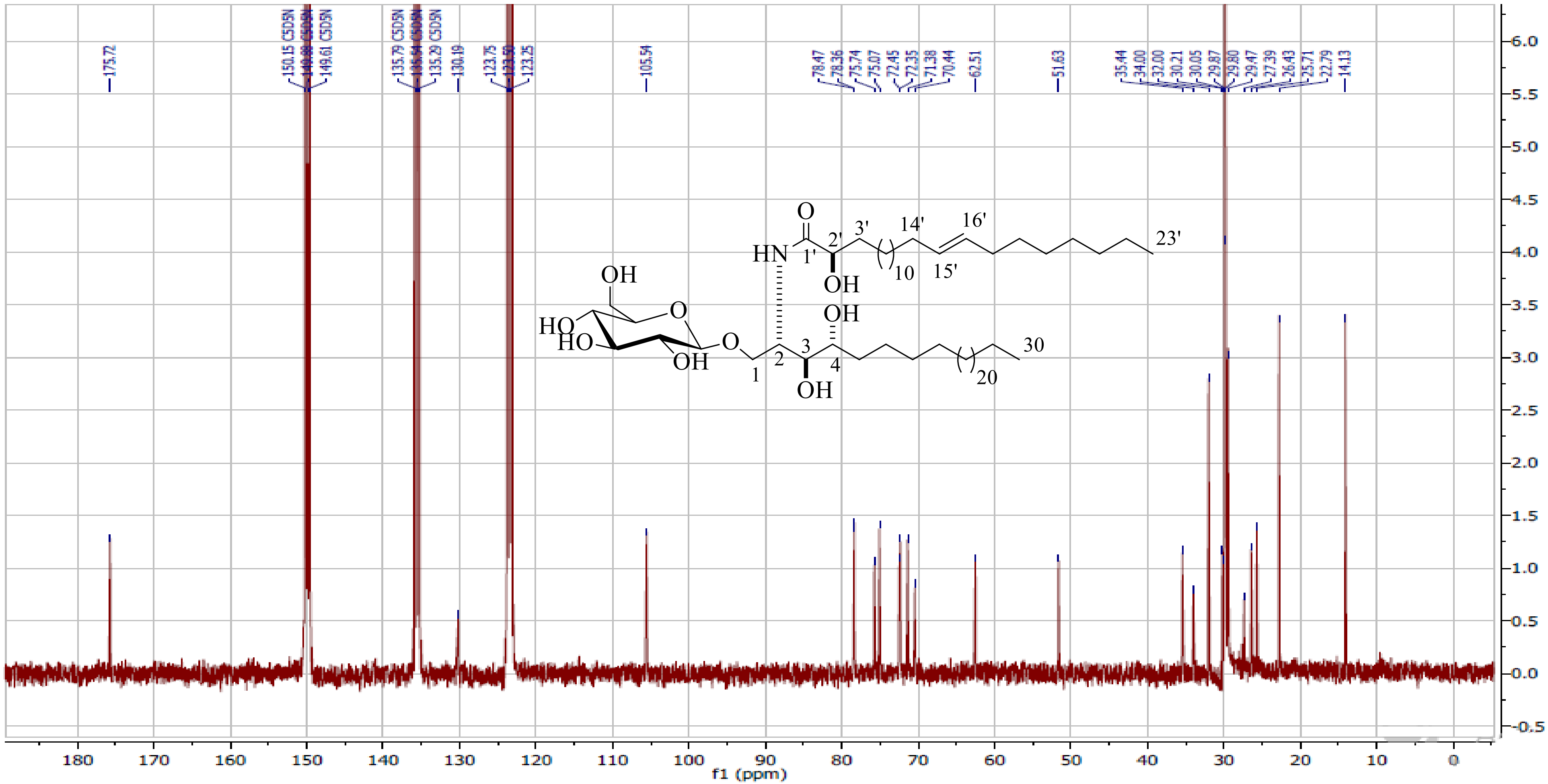

Figure S6: ^{13}C-NMR spectrum of compound 1 in (C_5D_5N, 100 MHz).

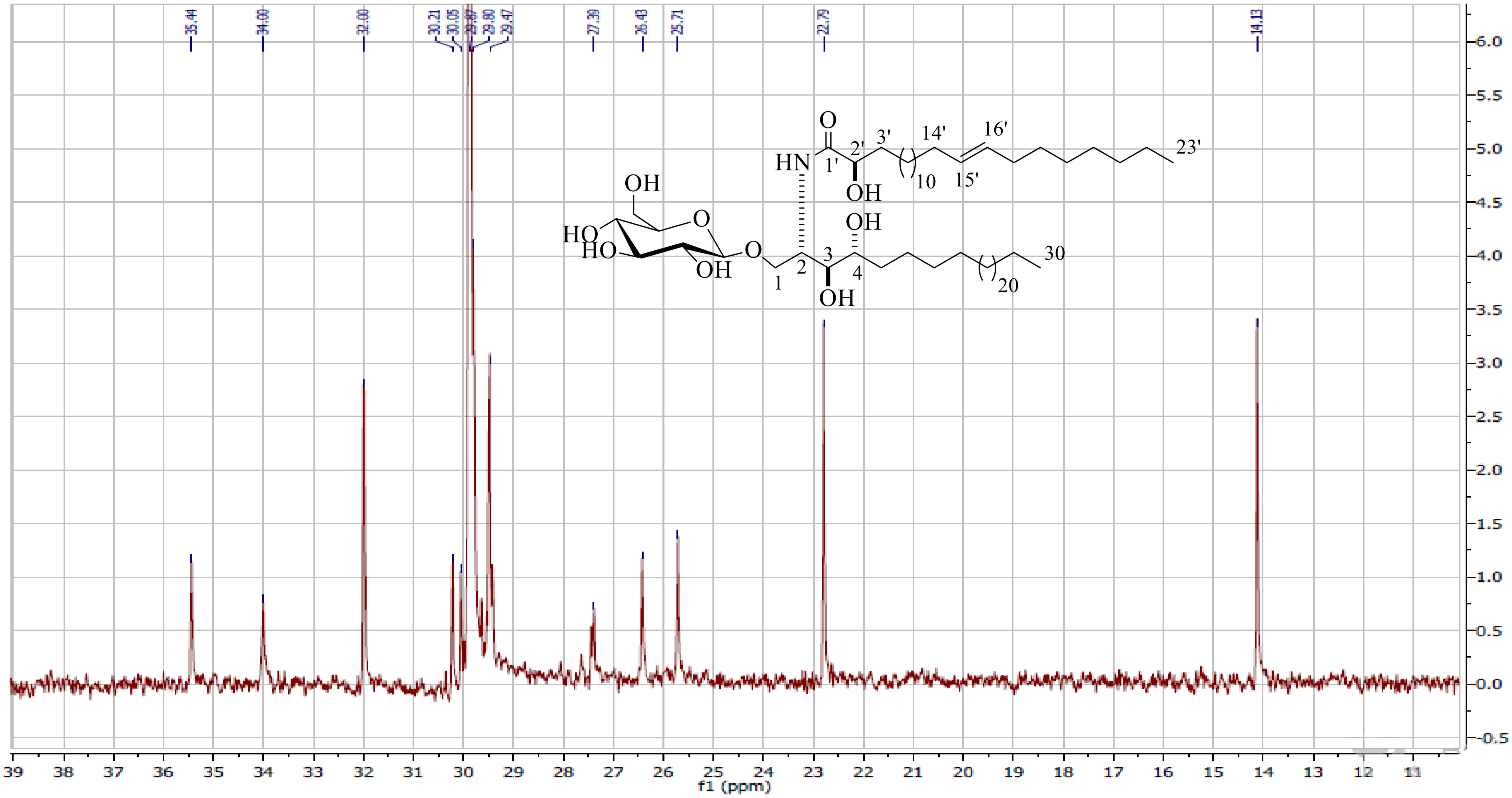

Figure S7: Partial expansion of the ^{13}C-NMR spectrum of compound 1 in (C_5D_5N, 100 MHz).

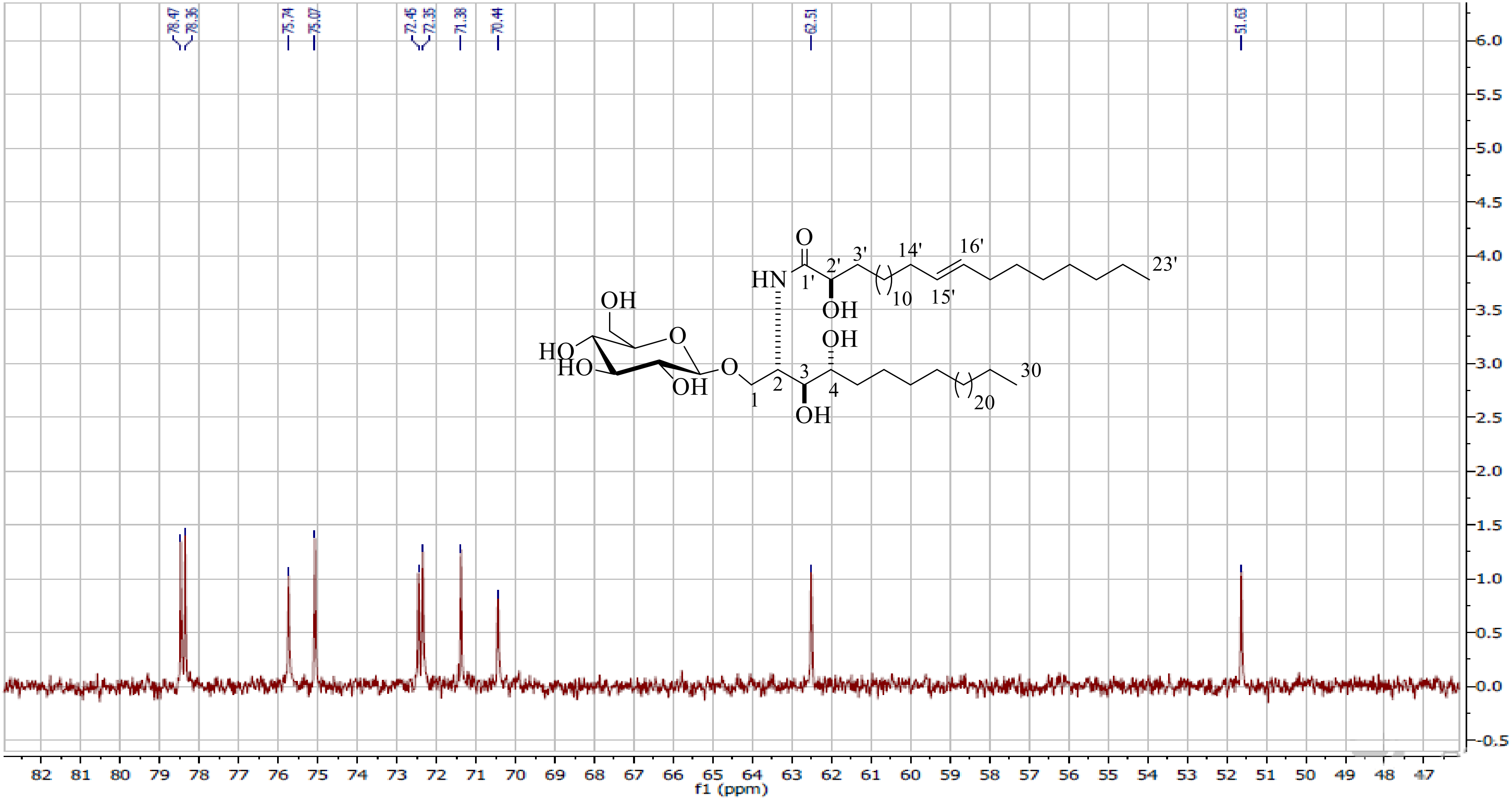

Figure S8: Partial expansion of the ^{13}C-NMR spectrum of compound 1 in (C_5D_5N, 100 MHz).

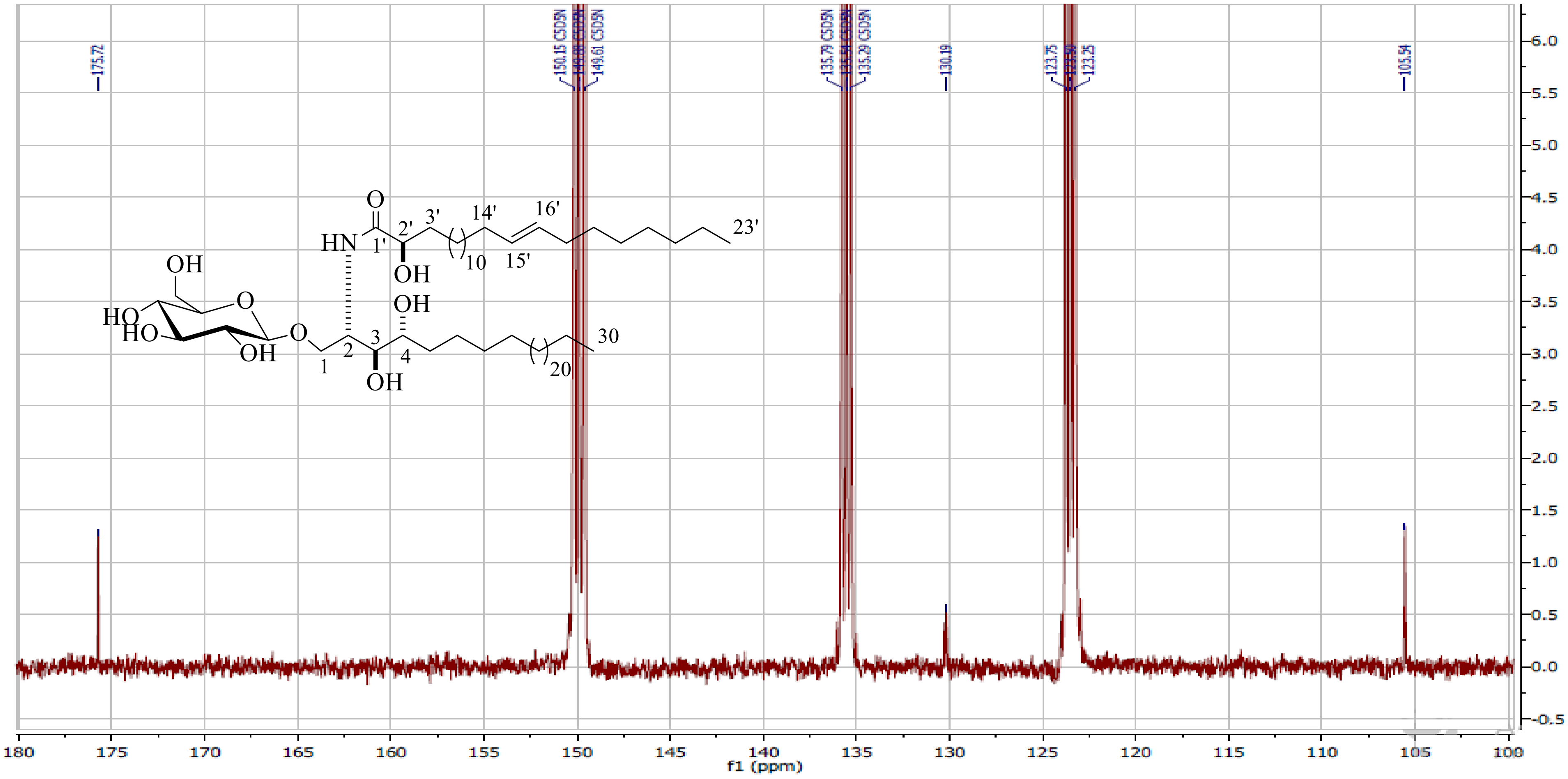

Figure S9: Partial expansion of the ^{13}C-NMR spectrum of compound 1 in (C_5D_5N, 100 MHz).

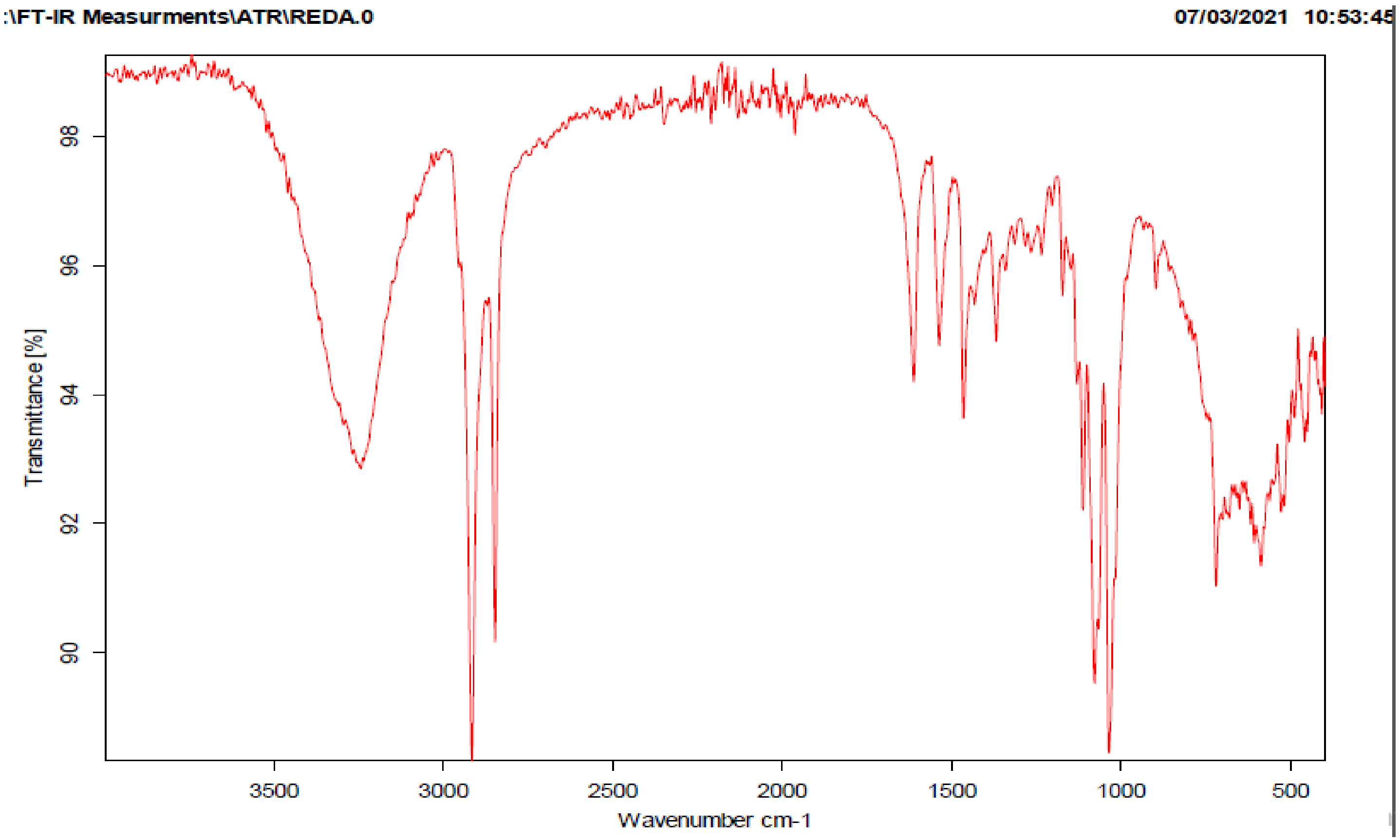

Figure S10: IR spectrum of compound 1.

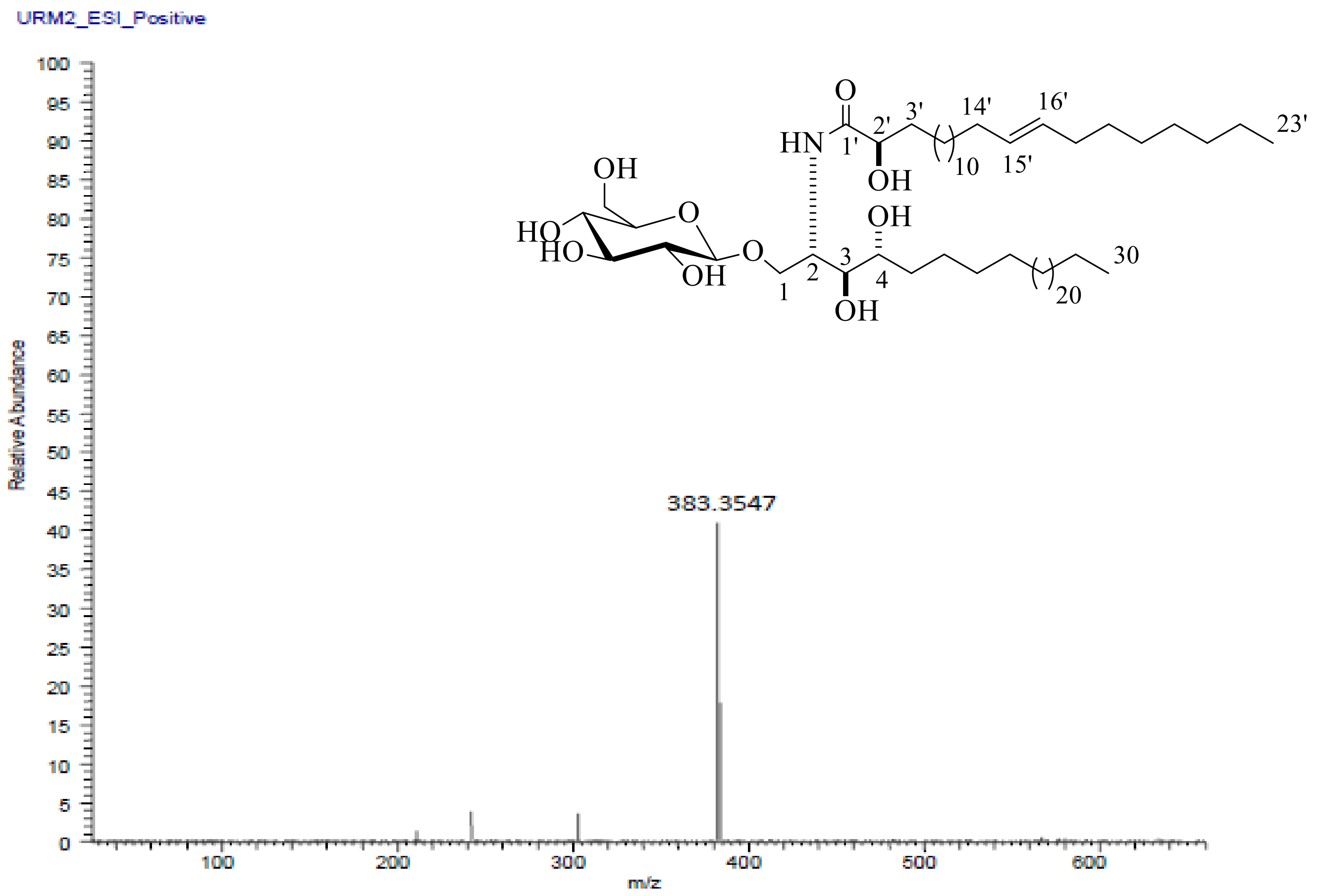

Figure S11: HRMS of *α*-hydroxy fatty acid methyl ester resulted from hydrolysis of compound 1

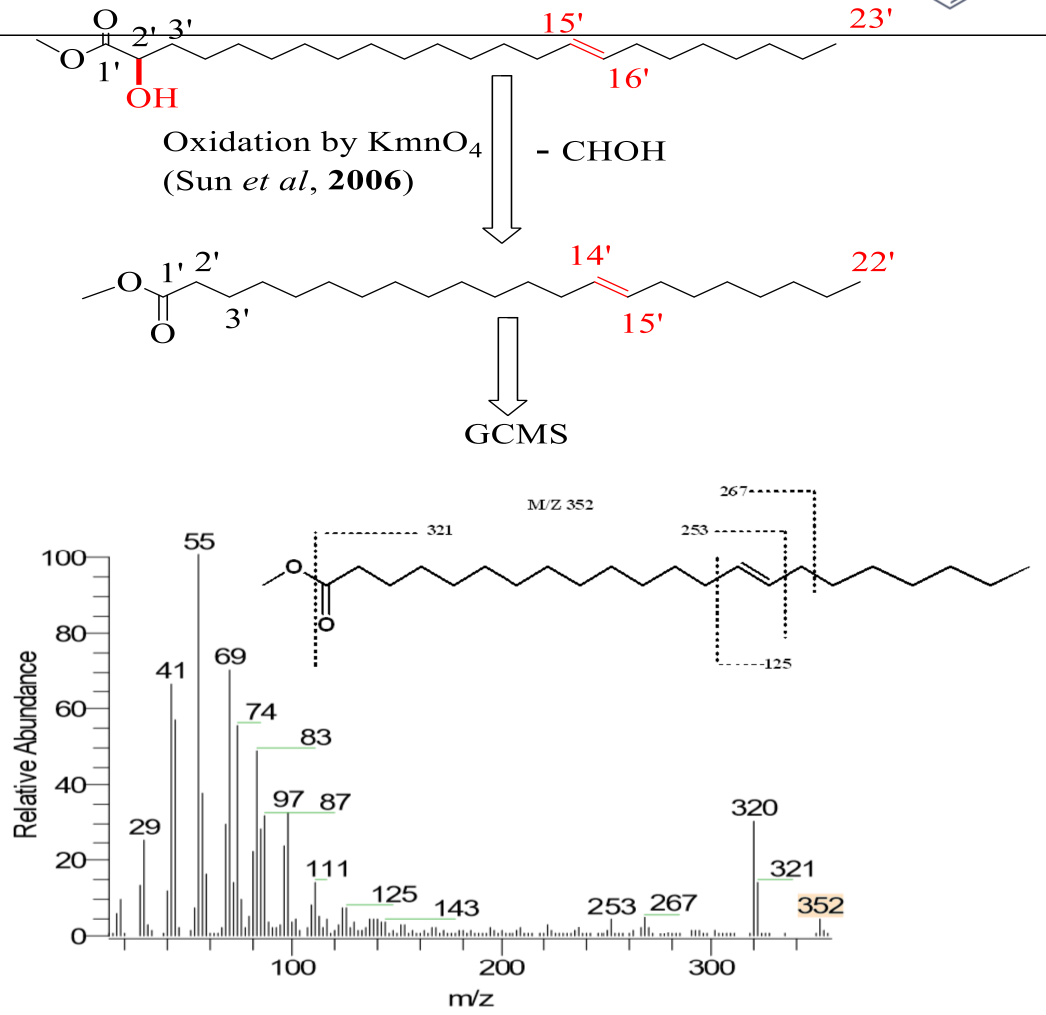

Figure S12: GC-MS analysis of fatty acids methyl ester carried out after oxidation of *α*-hydroxy fatty acid methyl ester (Compound 1)

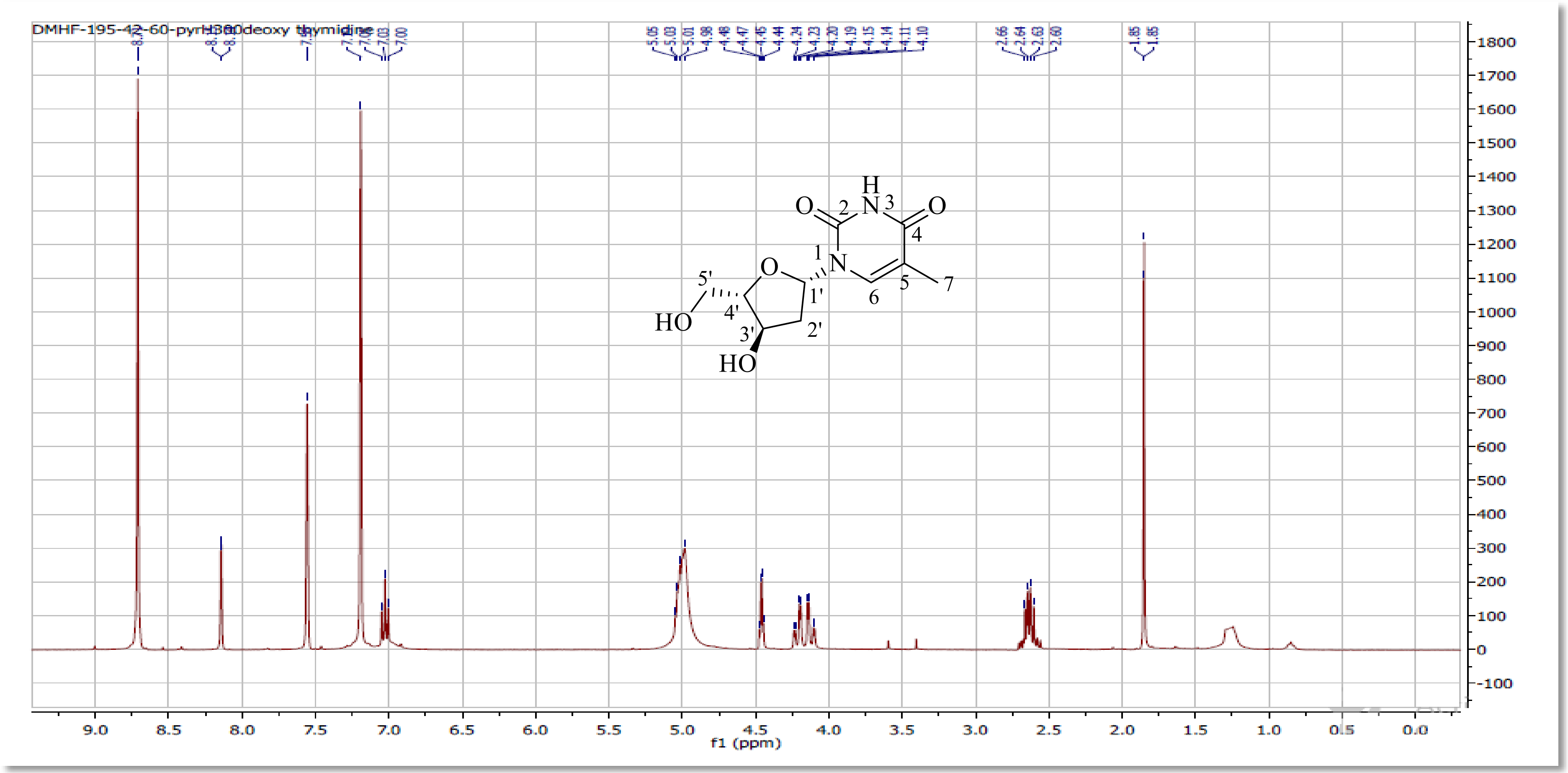

Figure S13: ^{1}H-NMR spectrum of compound 2 in (C_5D_5N, 400 MHz).

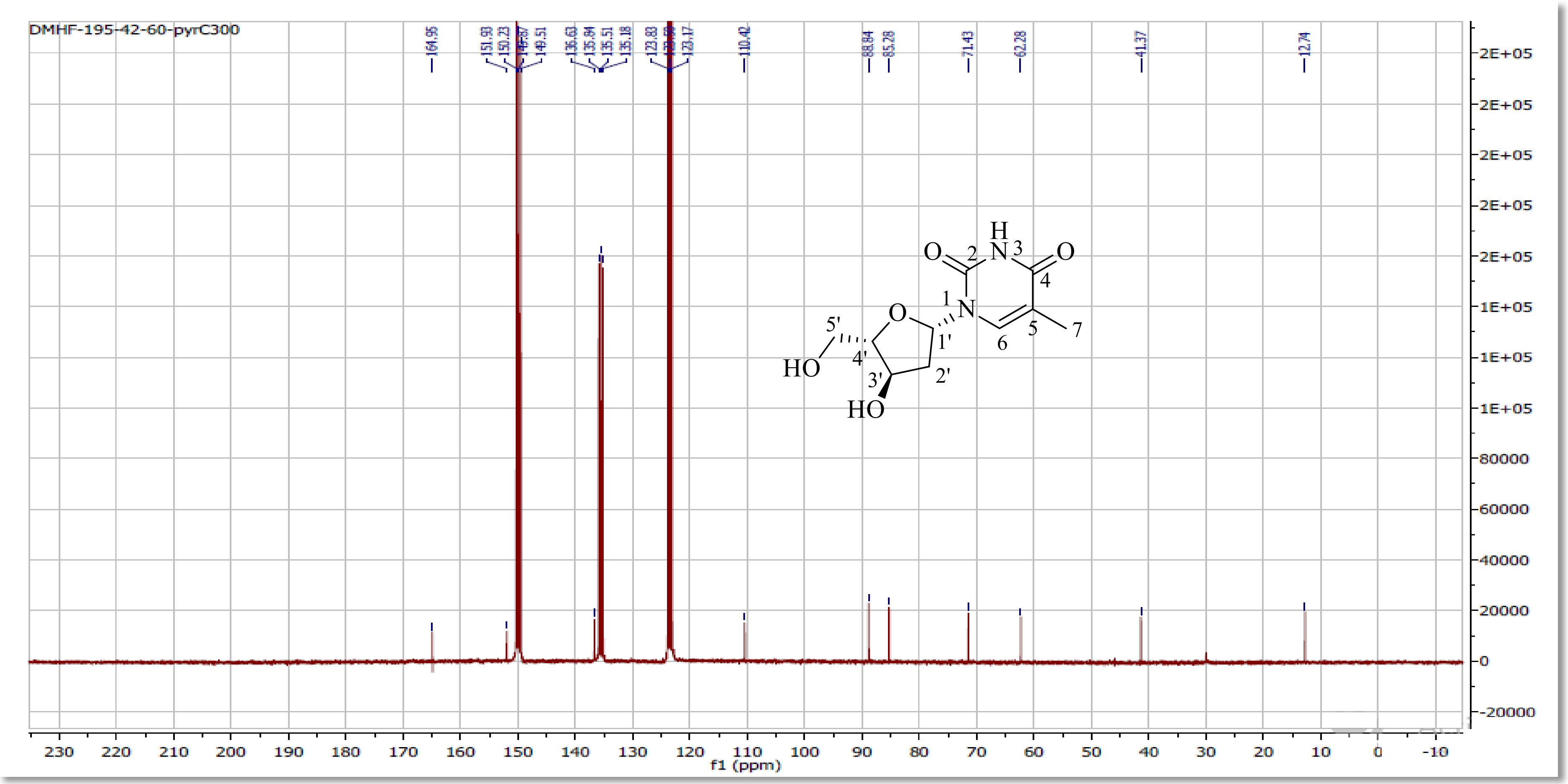

Figure S14: ^{13}C-NMR spectrum of compound 2 in (C_5D_5N, 100 MHz).

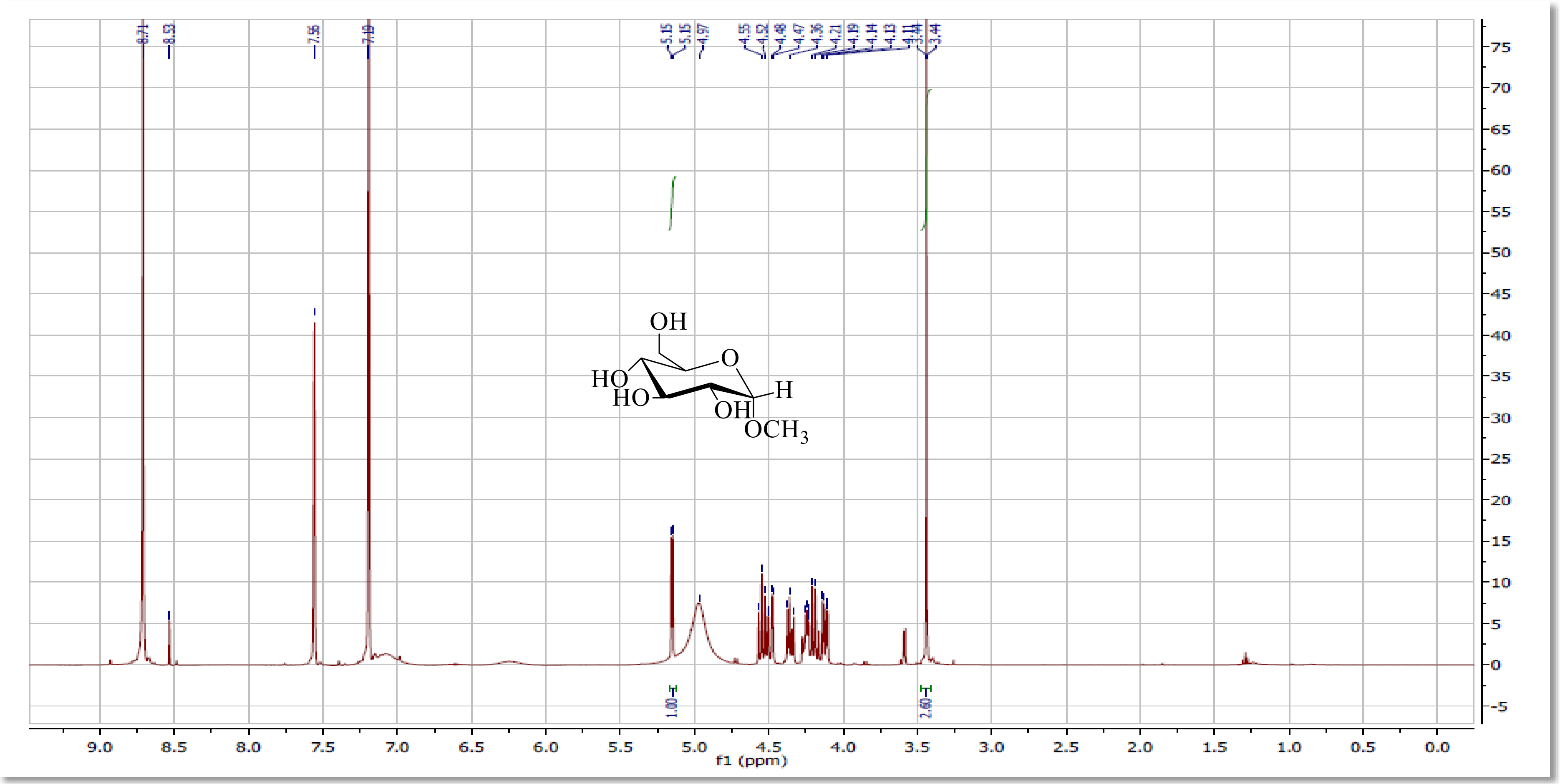

Figure S15: ^{1}H -NMR spectrum of Compound 3 in (C_5D_5N, 400 MHz).

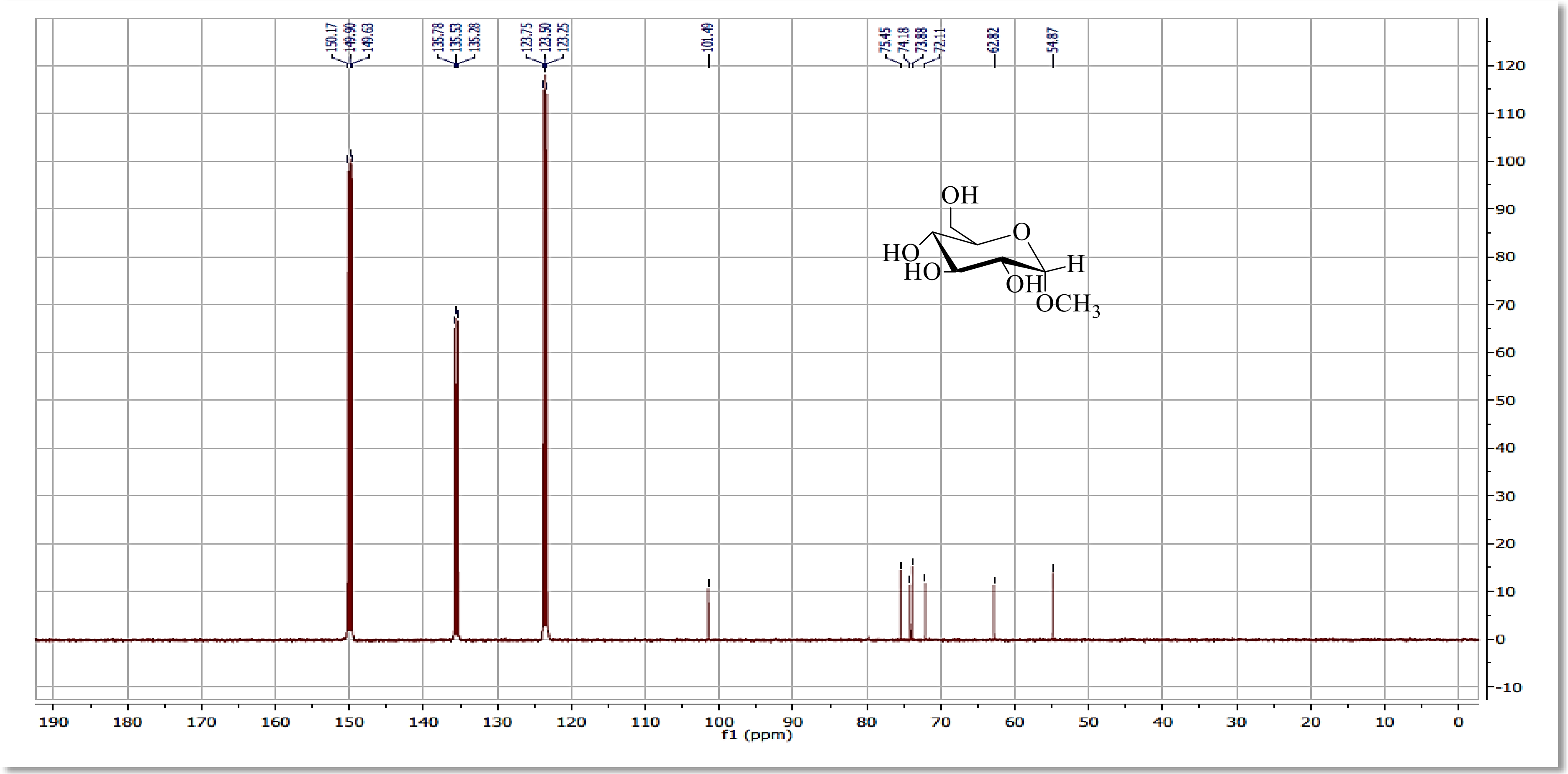

Figure S16: ^{13}C-NMR spectrum of Compound 3 (C_5D_5N, 100 MHz).

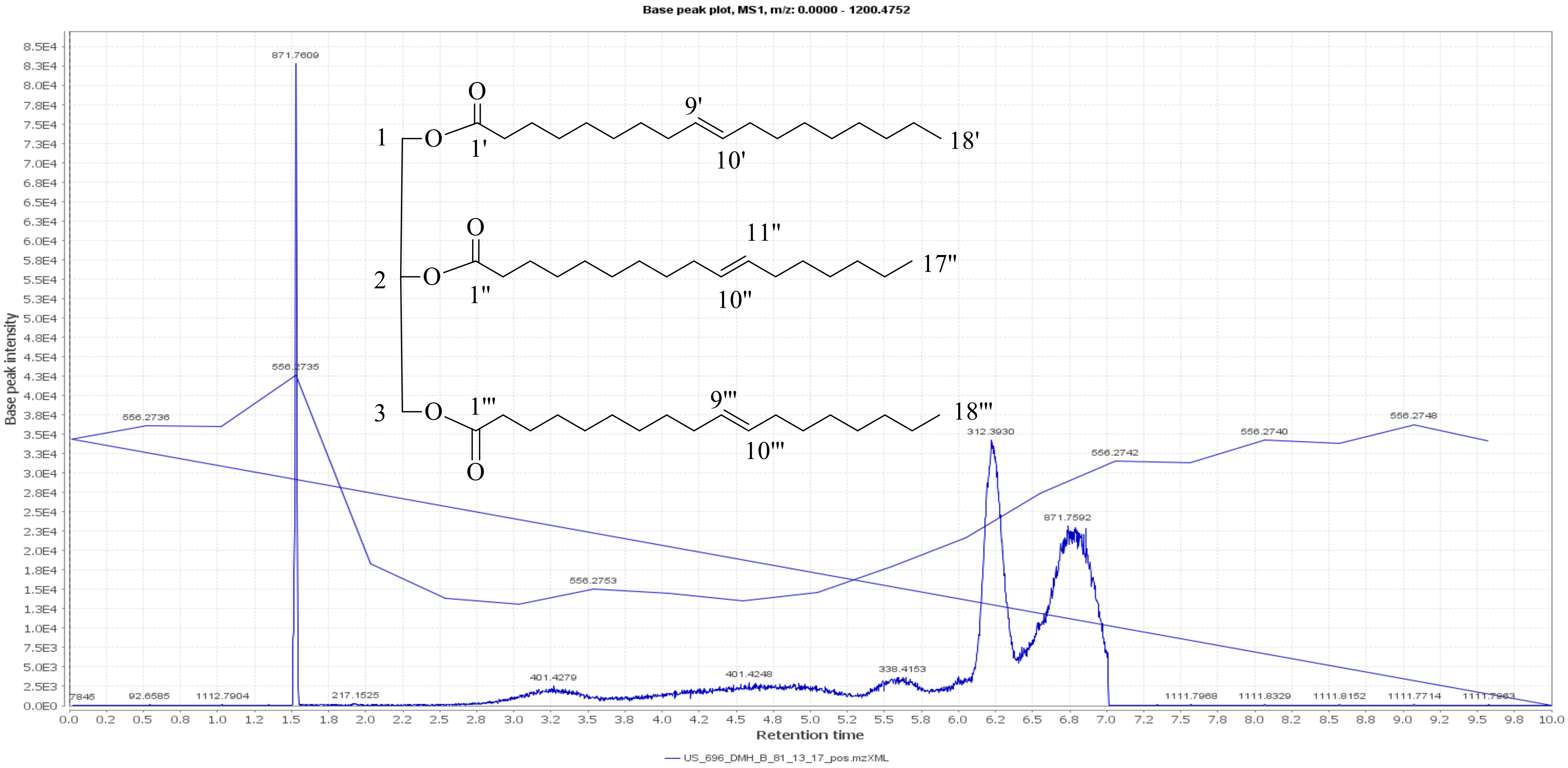

Figure S17: HRMS of Compound 4 (M+H)$^+$

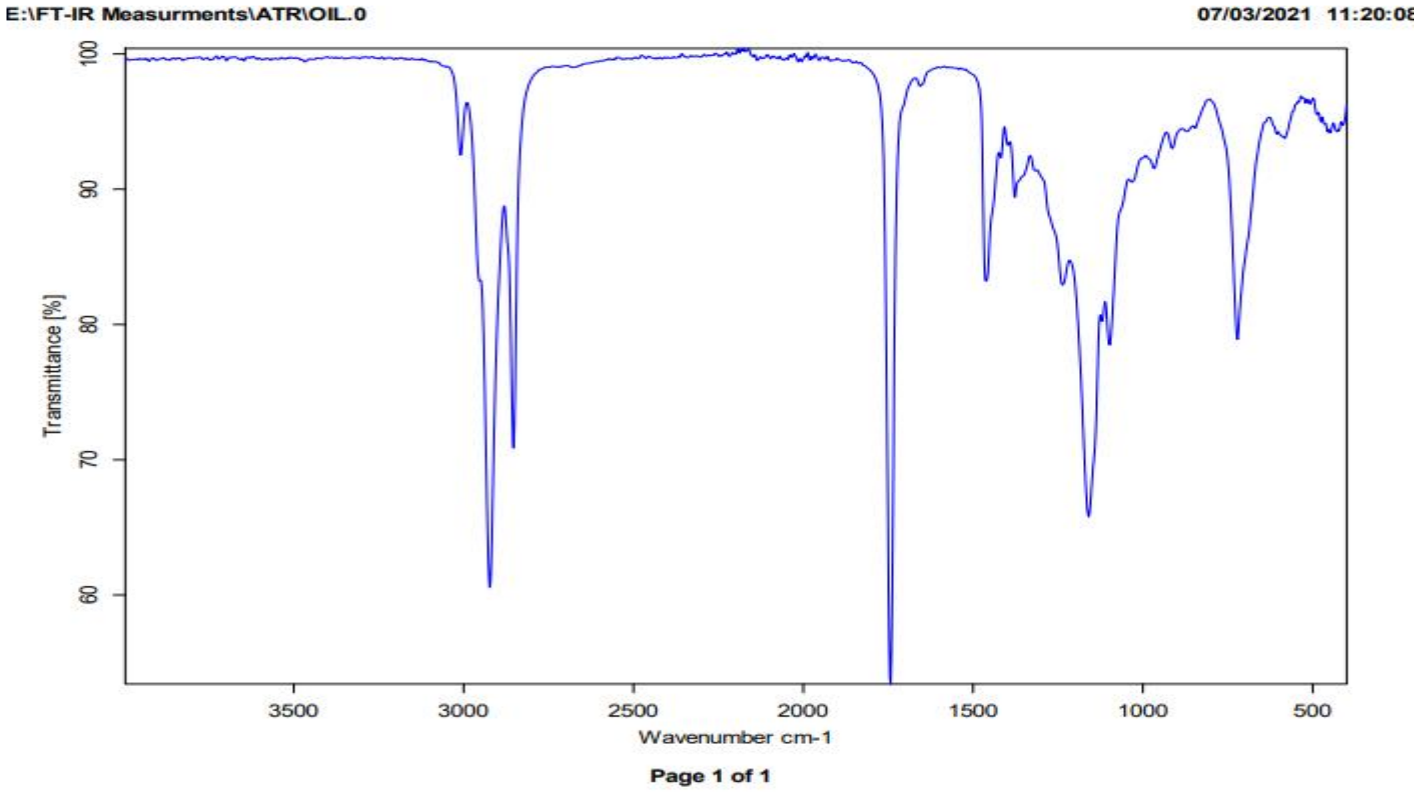
E:\FT-IR Measurments\ATR\OIL.0
07/03/2021 11:20:08
Transmittance [%]
100
90
80
70
60
3500
3000
2500
2000
1500
1000
500
Wavenumber cm-1
Page 1 of 1

Figure 16

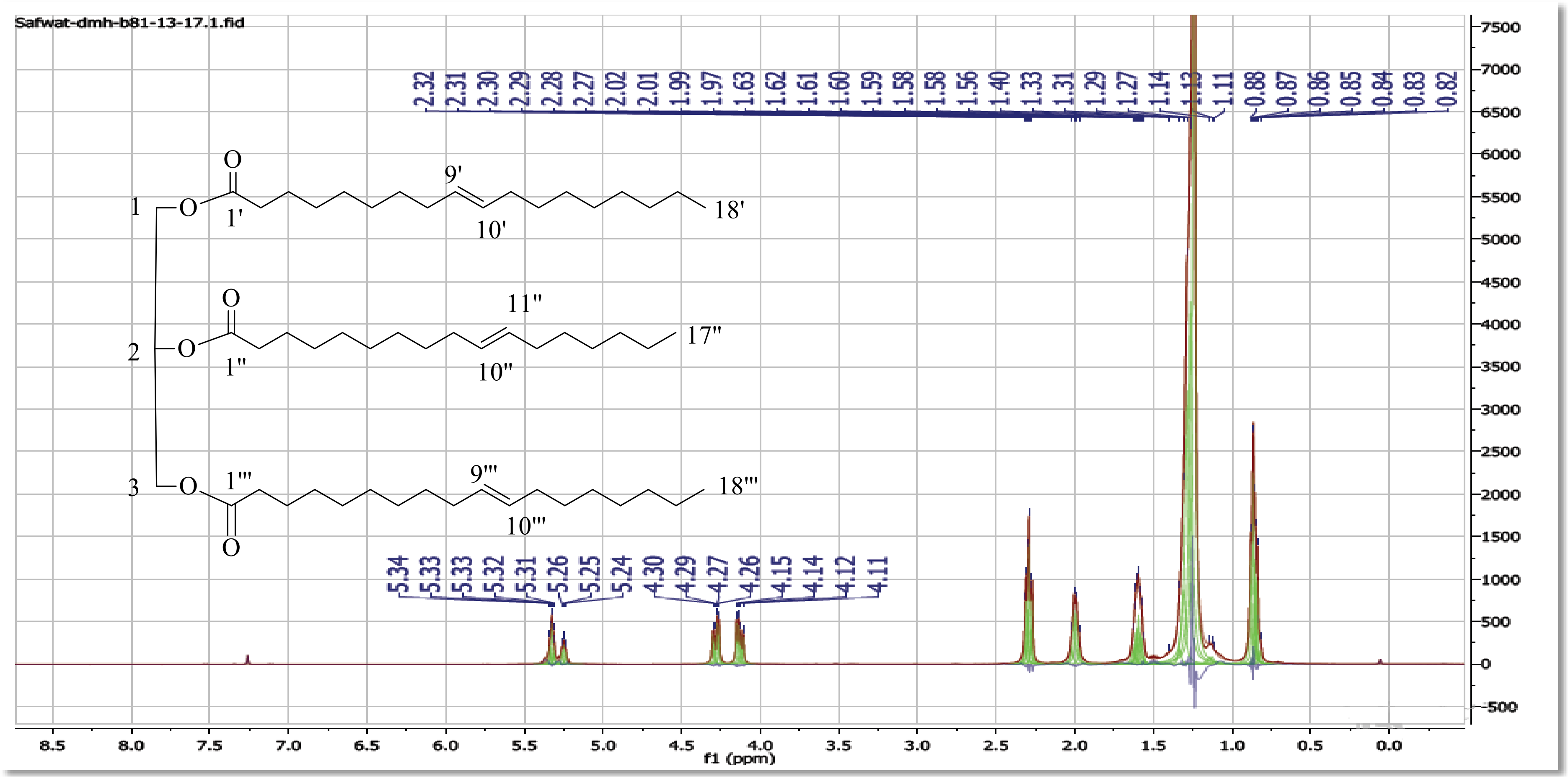

Figure S19: ^{1}H-NMR spectrum of compound 4 in ($CDCl_3$, 400 MHz).

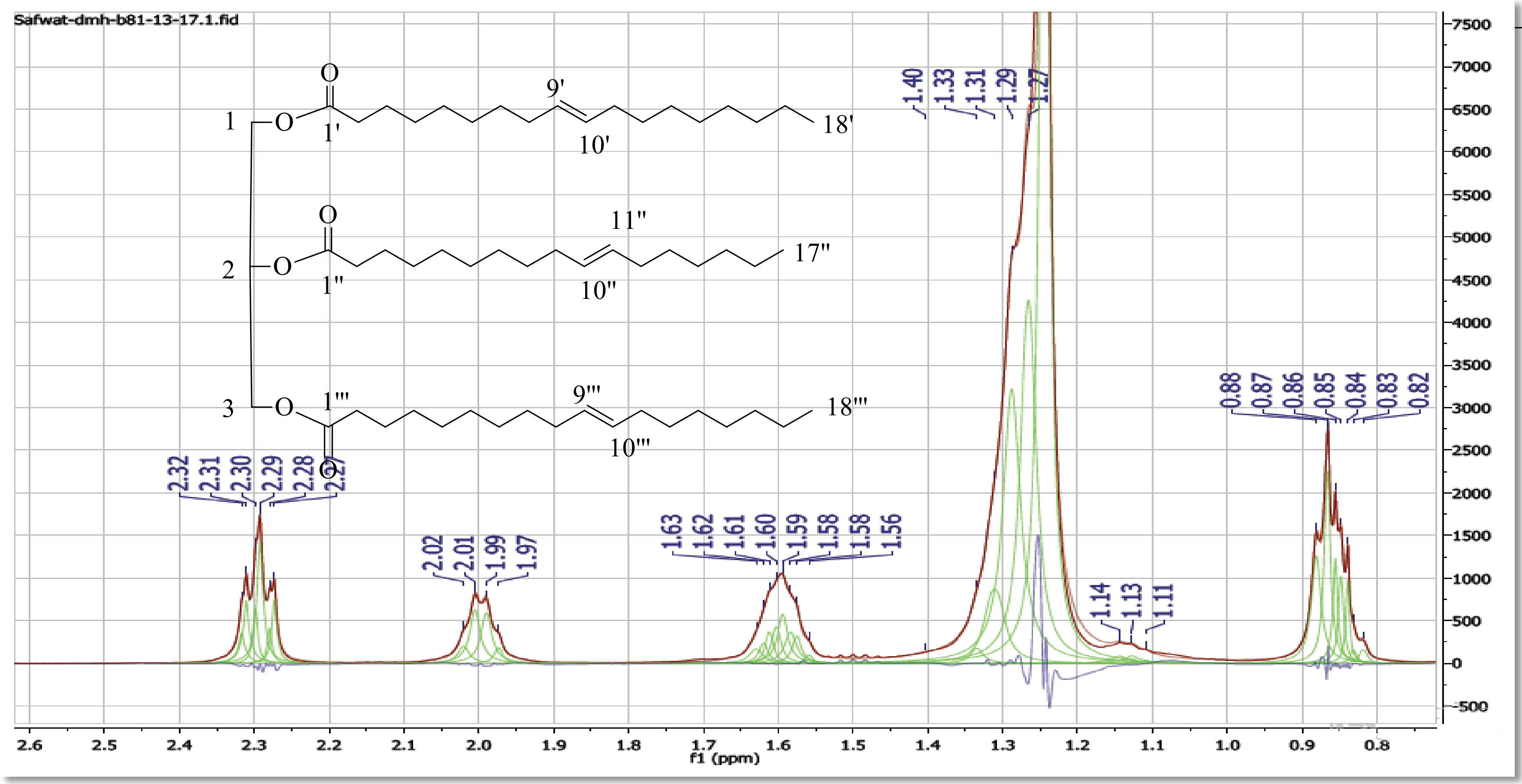

Figure S20: Partial expansions of the ^{1}H-NMR spectrum of compound 4 in ($CDCl_3$, 400 MHz)

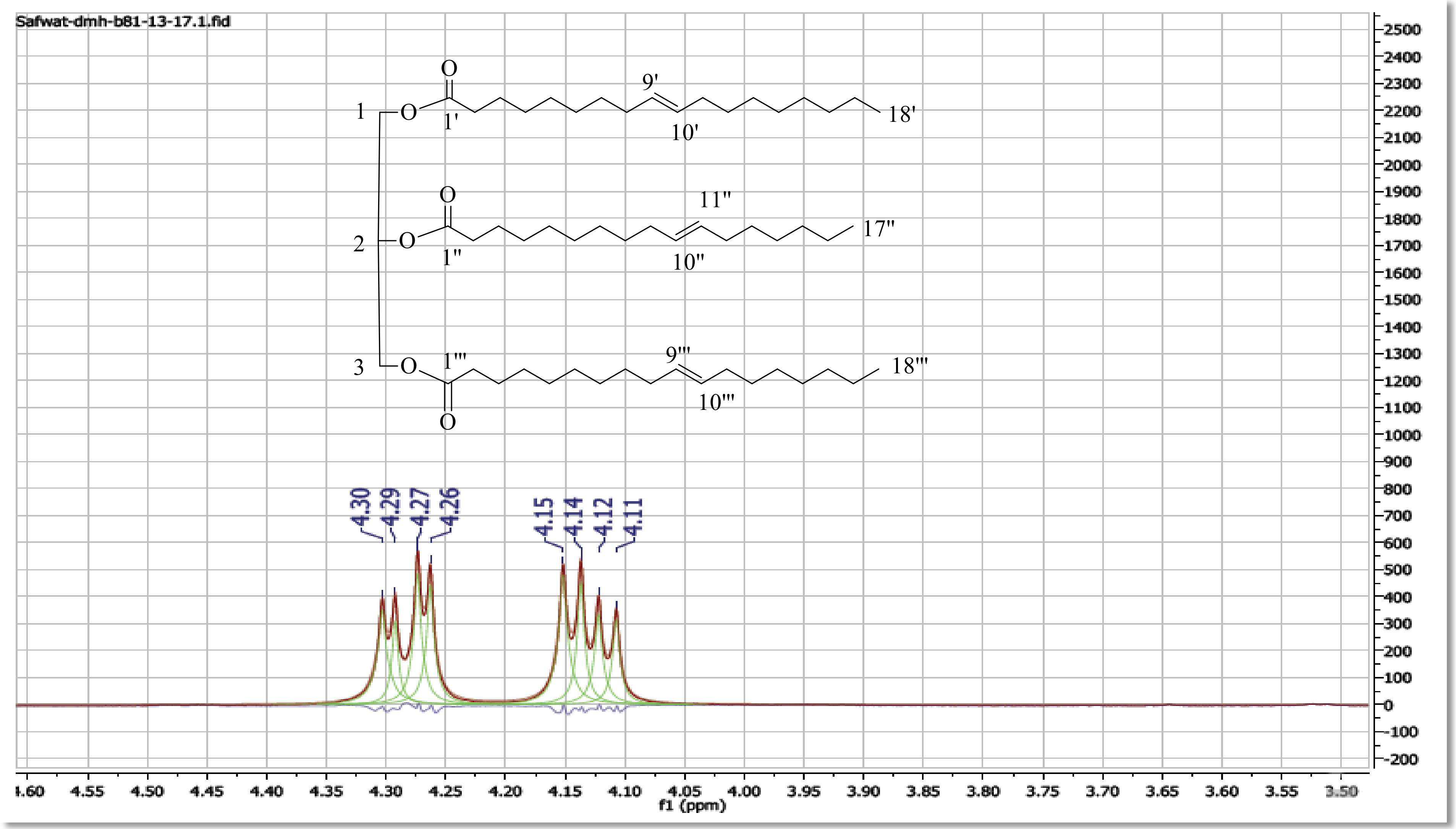

Figure S21: Partial expansions of the ^{1}H-NMR spectrum of compound 4 in ($CDCl_3$, 400 MHz)

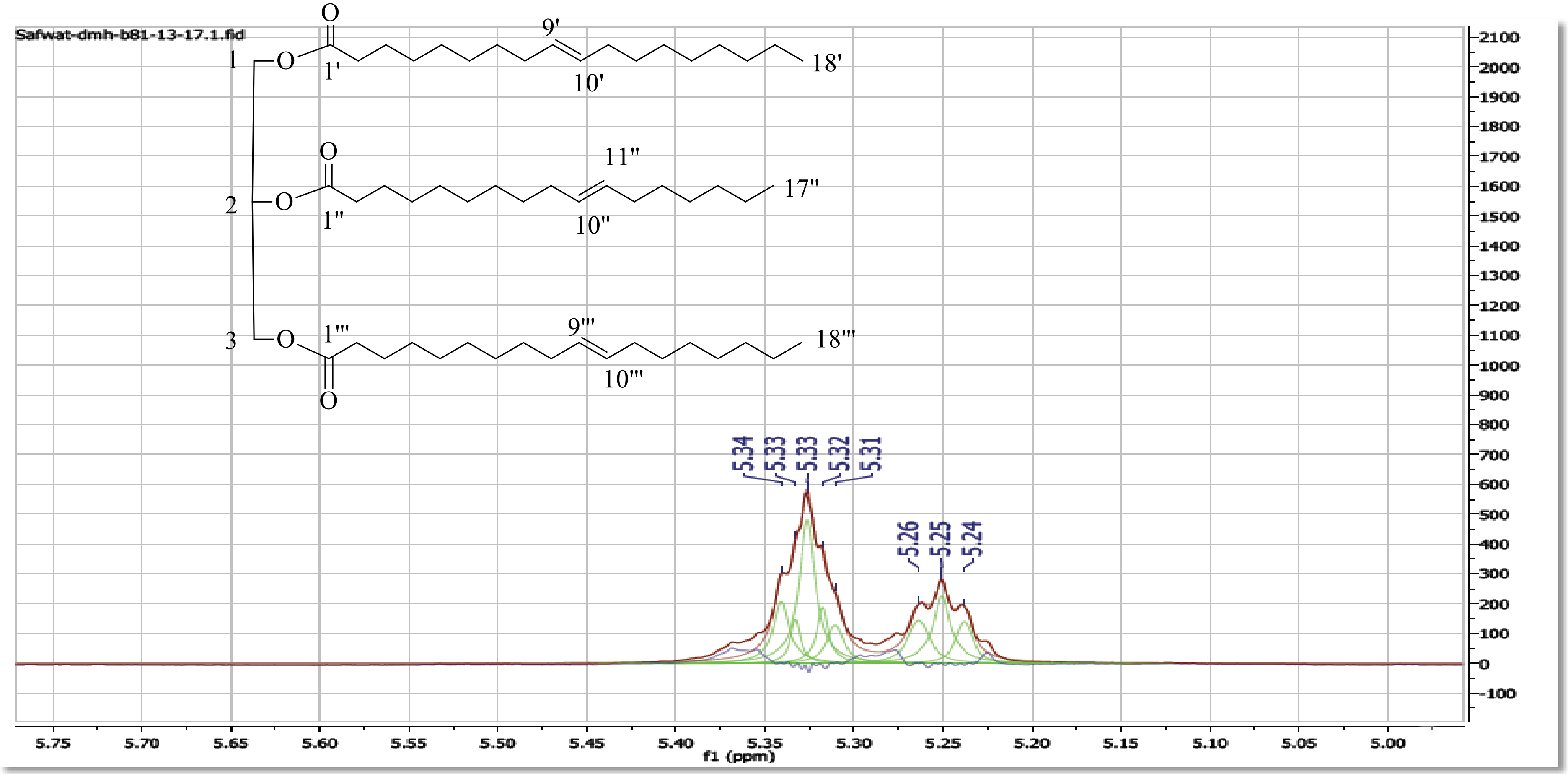

Figure S22: Partial expansions of the ^{1}H-NMR spectrum of compound 4 in ($CDCl_3$, 400 MHz)

molecules

MDPI

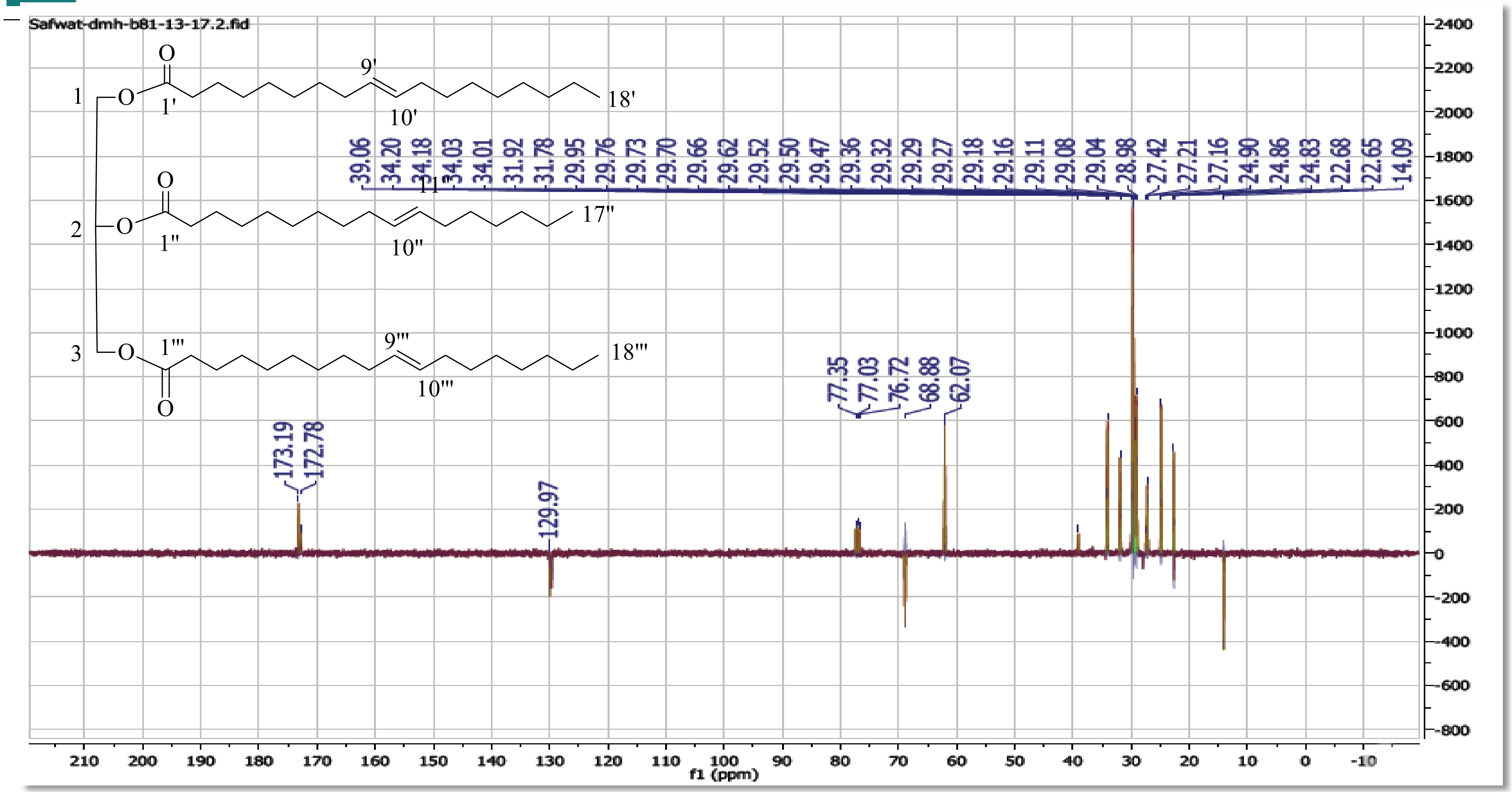

Figure S23: ^{13}C-NMR spectrum of compound 4 in (CDCl$_3$, 400 MHz).

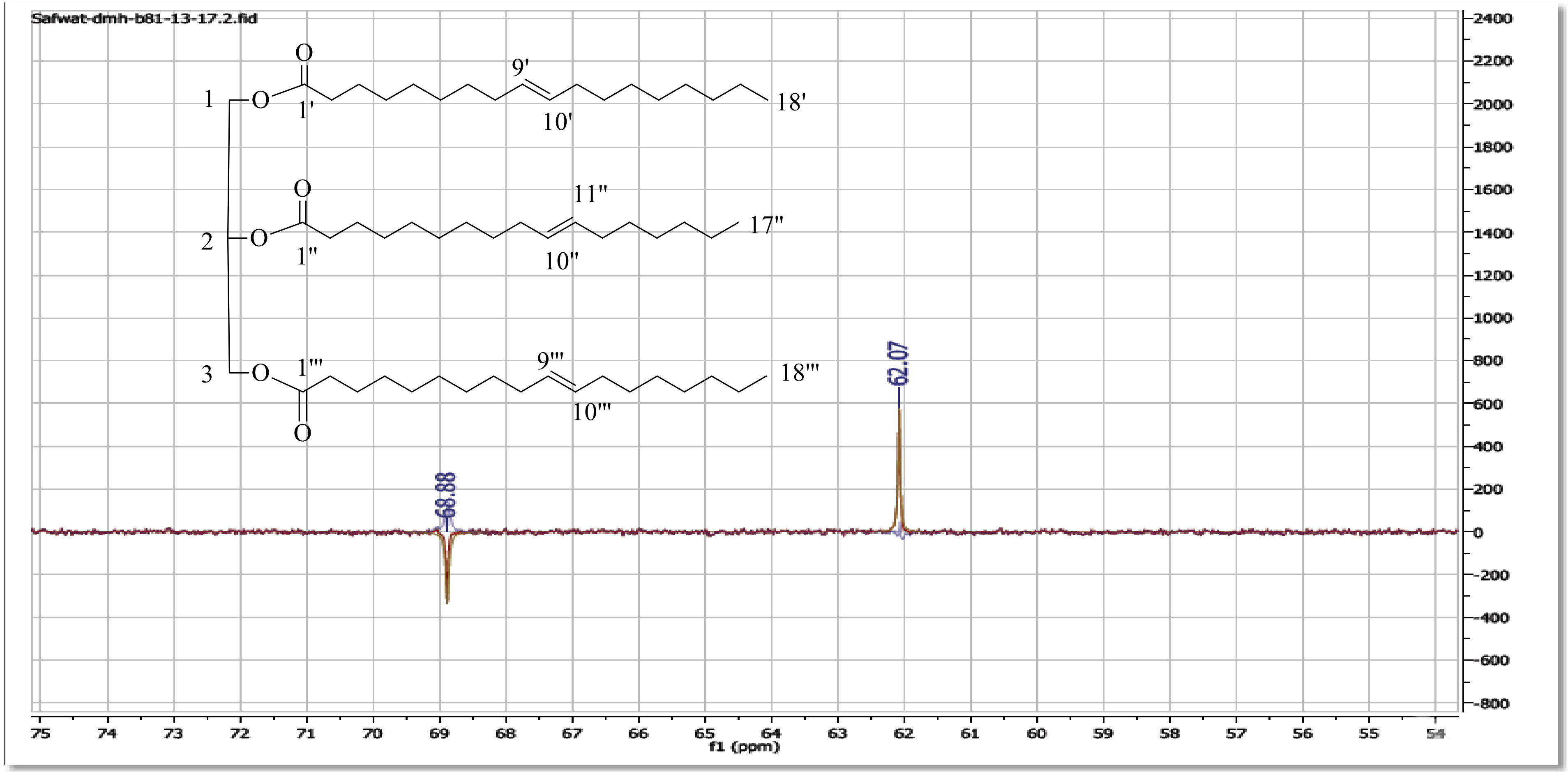

Figure S24: Partial expansion ^{13}C-NMR spectrum of compound 4 in (CDCl$_3$, 400 MHz).

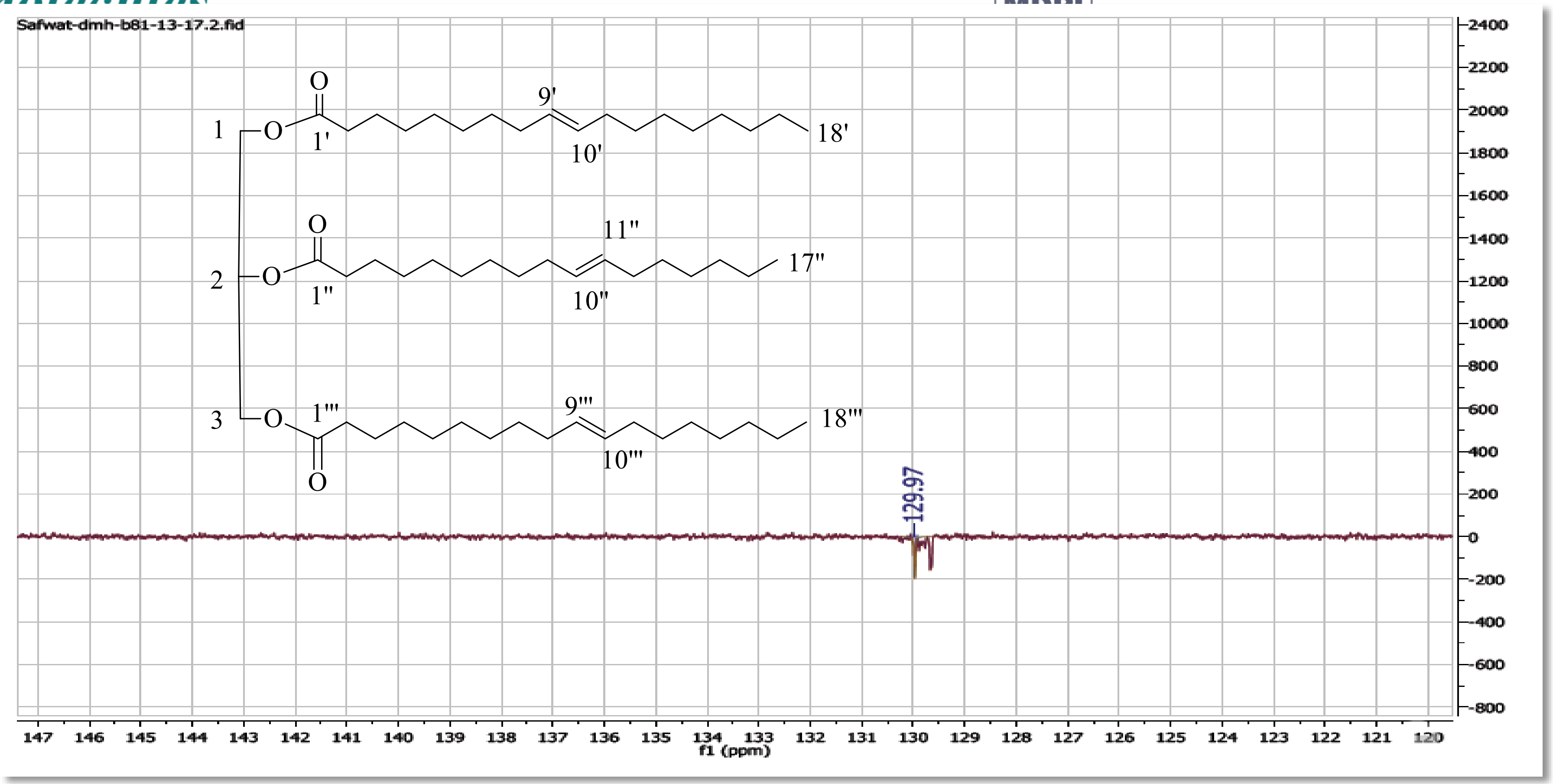

Figure S25: Partial expansion ^{13}C-NMR spectrum of compound 4 in ($CDCl_3$, 400 MHz).

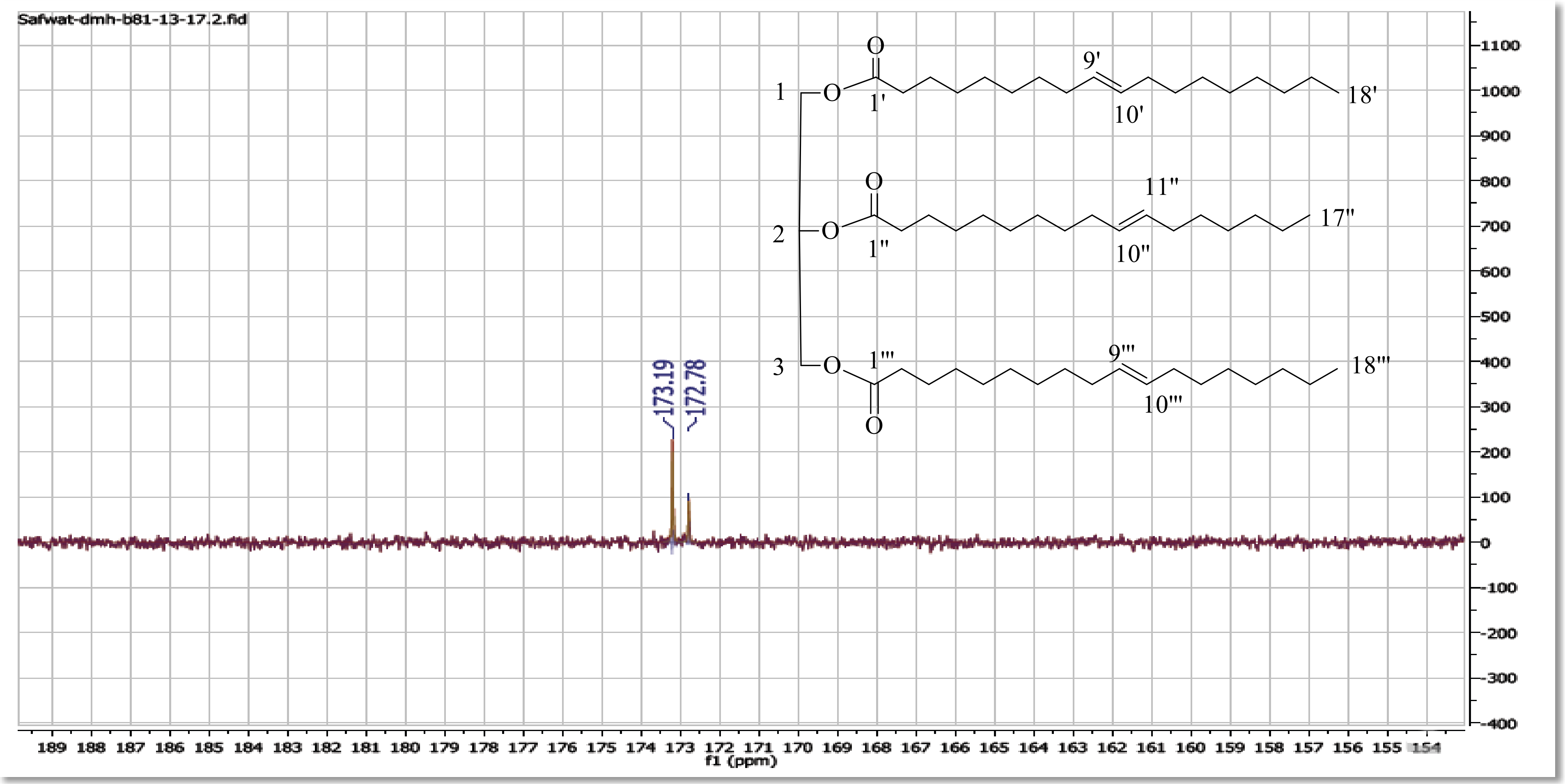

Figure S26: Partial expansion ^{13}C-NMR spectrum of compound 4 in ($CDCl_3$, 400 MHz).

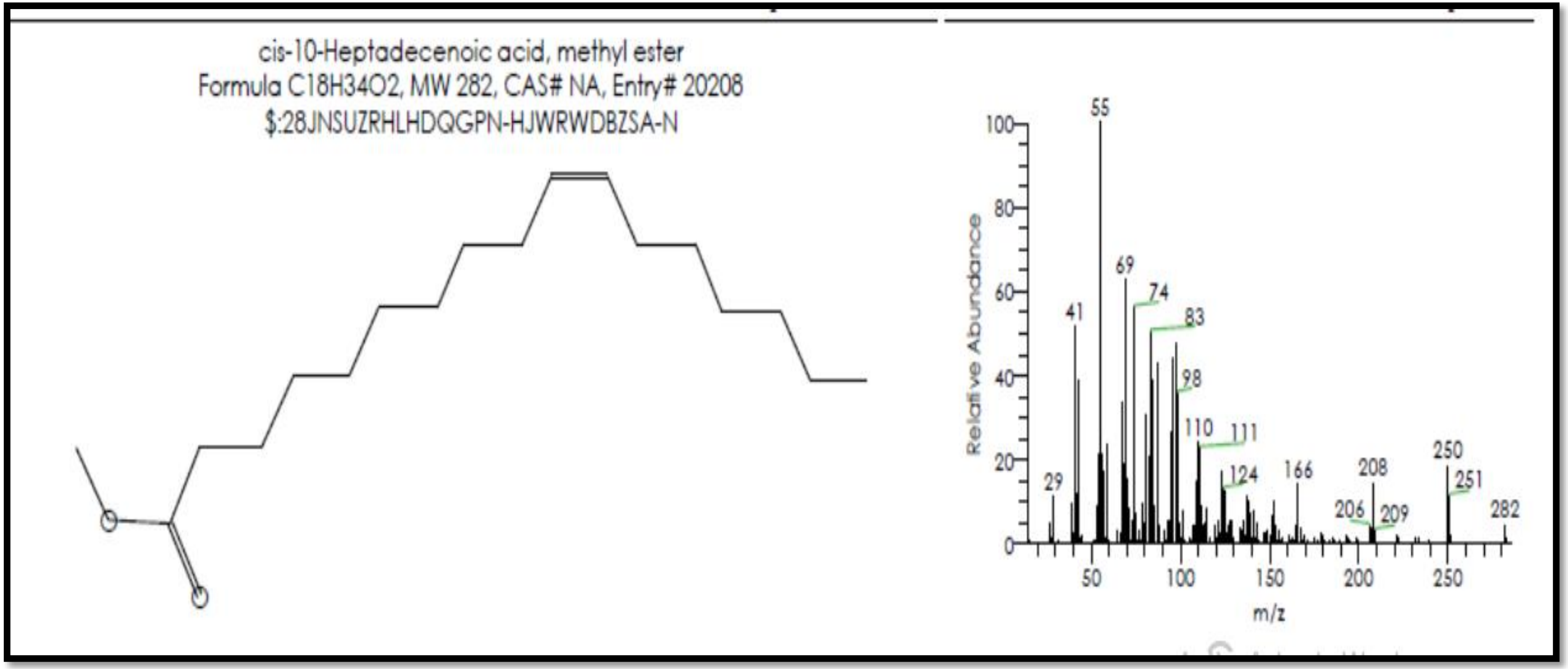

Figure S27: MS spectrum for 10-Heptadecenoic acid (z)- methyl ester.

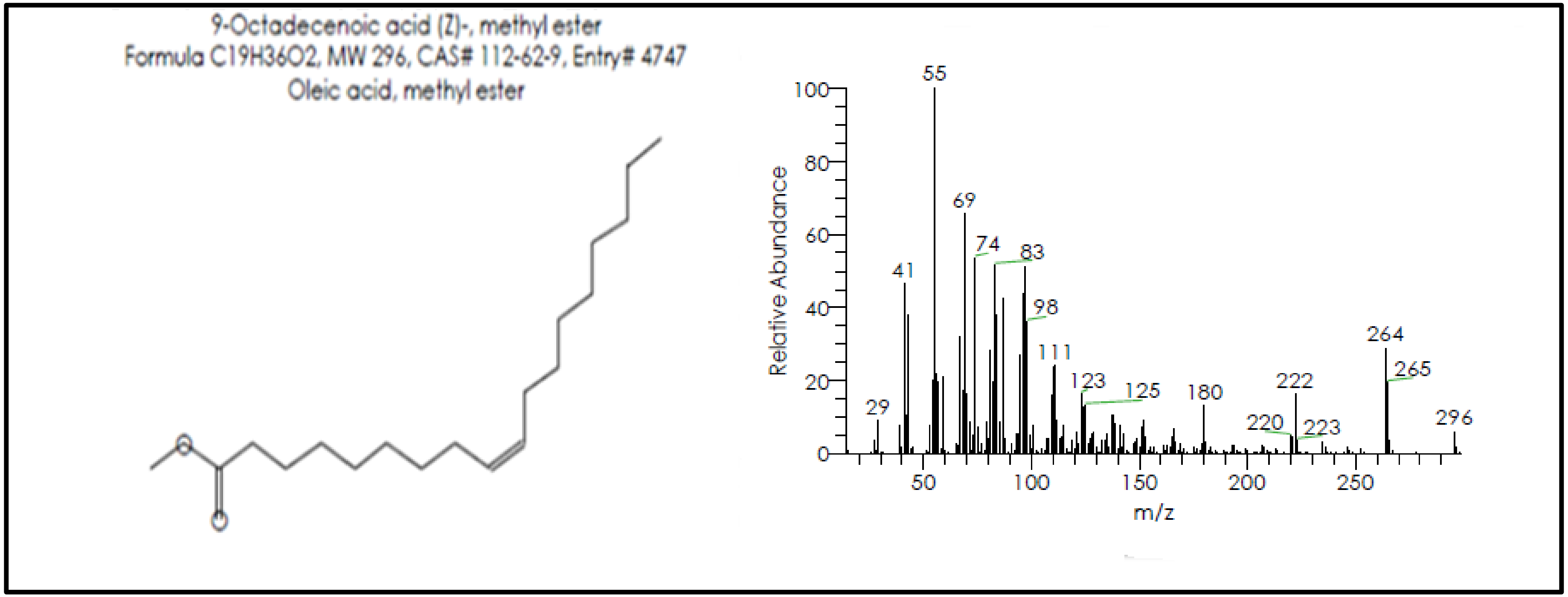

Figure S28: MS spectrum for 9-Octadecenoic acid (z)-methyl ester

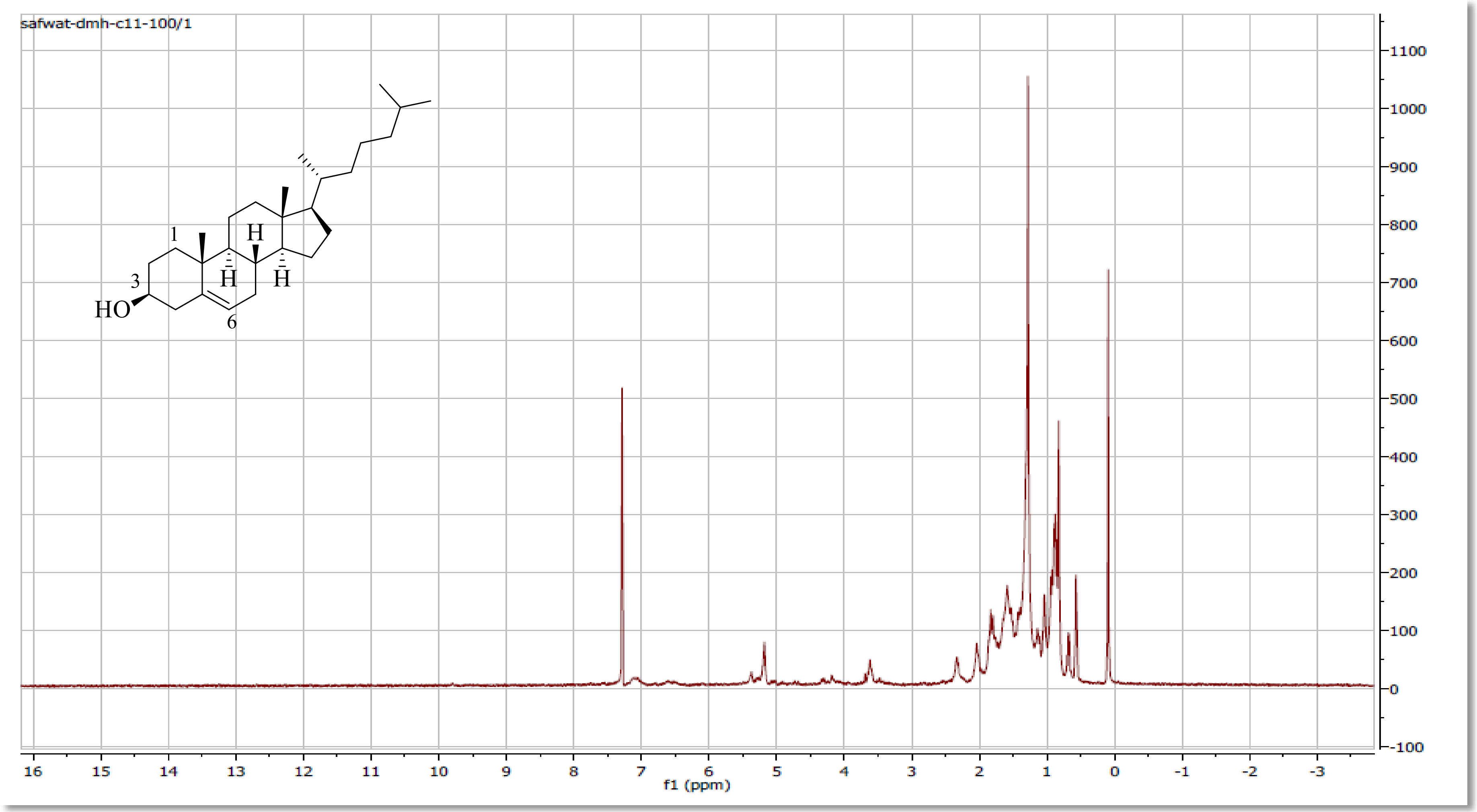

Figure S29: ^{1}H -NMR spectrum of Compound 5 in ($CDCl_3$, 400 MHz).

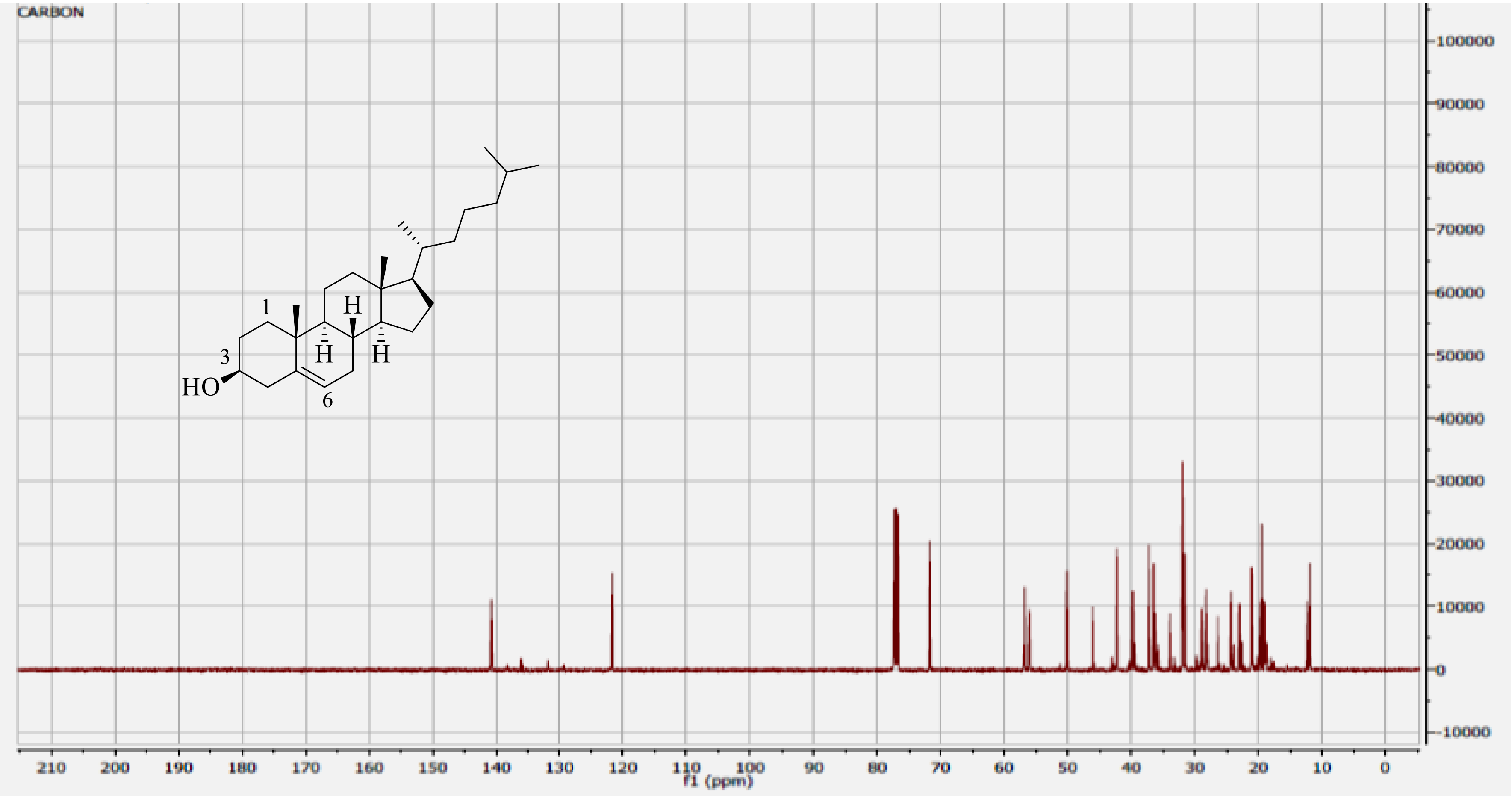

Figure S30: ^{13}C-NMR spectrum of Compound 5 ($CDCl_3$, 100 MHz).

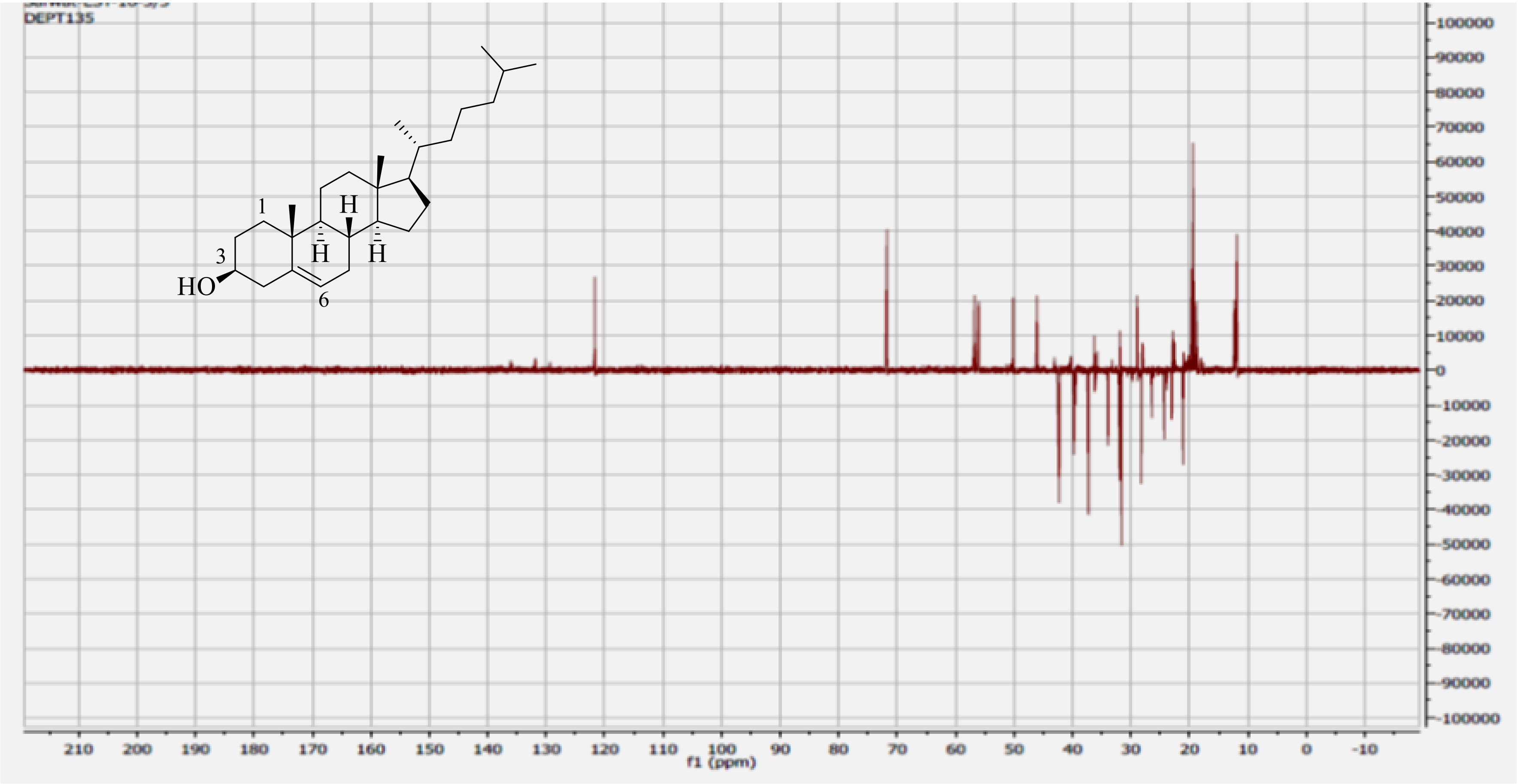

Figure S31: DEPT spectrum of Compound 5 in ($CDCl_3$, 100 MHz).
